# Design, Synthesis and In Vitro Evaluation of Spirooxindole-Based Phenylsulfonyl Moiety as a Candidate Anti-SAR-CoV-2 and MERS-CoV-2 with the Implementation of Combination Studies

**DOI:** 10.3390/ijms231911861

**Published:** 2022-10-06

**Authors:** Assem Barakat, Ahmed Mostafa, M. Ali, Abdullah Mohammed Al-Majid, Luis R. Domingo, Omnia Kutkat, Yassmin Moatasim, Komal Zia, Zaheer Ul-Haq, Yaseen A. M. M. Elshaier

**Affiliations:** 1 Department of Chemistry, College of Science, King Saud University, P.O. Box 2455, Riyadh 11451, Saudi Arabia; 2 Center of Scientific Excellence for Influenza Viruses, National Research Centre, Giza 12622, Egypt; 3 Department of Organic Chemistry, University of Valencia, Dr. Moliner 50, 46100 Burjassot, Valencia, Spain; 4 Dr. Panjwani Center for Molecular Medicine and Drug Research, International Center for Chemical and Biological Sciences, University of Karachi, Karachi 75270, Pakistan; 5 H.E.J. Research Institute of Chemistry, International Center for Chemical and Biological Sciences, University of Karachi, Karachi 75270, Pakistan; 6 Department of Organic and Medicinal Chemistry, Faculty of Pharmacy, University of Sadat City, Menoufiya 32958, Egypt

**Keywords:** spirooxindole, SARS-CoV-2, drug combination protocol, molecular dynamic simulation (MDS), ADMET

## Abstract

The search for an effective anti-viral to inhibit COVID-19 is a challenge for the specialized scientific research community. This work investigated the anti-coronavirus activity for spirooxindole-based phenylsulfone cycloadducts in a single and combination protocols. The newly designed anti-SARS-CoV-2 therapeutics spirooxindoles synthesized by [3 + 2] cycloaddition reactions represent an efficient approach. One-pot multicomponent reactions between phenyl vinyl sulfone, substituted isatins, and amines afforded highly stereoselective anti-SARS-CoV-2 therapeutics spirooxindoles with three stereogenic centers. Herein, the newly synthesized spirooxindoles were assessed individually against the highly pathogenic human coronaviruses and proved to be highly potent and safer. Interestingly, the synergistic effect by combining the potent, tested spirooxindoles resulted in an improved antiviral activity as well as better host-cell safety. Compounds **4i** and **4d** represented the most potent activity against MERS-CoV with IC_50_ values of 11 and 23 µM, respectively. Both compounds **4c** and **4e** showed equipotent activity with the best IC_50_ against SARS-CoV-2 with values of 17 and 18 µM, respectively, then compounds **4d** and **4k** with IC_50_ values of 24 and 27 µM, respectively. Then, our attention oriented to perform a combination protocol as anti-SARS-CoV-2 for the best compounds with a different binding mode and accompanied with different pharmacophores. Combination of compound **4k** with **4c** and combination of compounds **4k** with **4i** proved to be more active and safer. Compounds **4k** with **4i** displayed IC_50_ = 3.275 µM and half maximal cytotoxic-concentration CC_50_ = 11832 µM. MD simulation of the most potential compounds as well as in silico ADMET properties were investigated. This study highlights the potential drug-like properties of spirooxindoles as a cocktail anti-coronavirus protocol.

## 1. Introduction

Since 2012, the world has been confronted with two highly pathogenic coronaviruses including the Middle East respiratory syndrome coronavirus (MERS-CoV) and severe acute respiratory syndrome coronavirus 2 (SARS-CoV-2) [1,2]. MERS is a viral respiratory infection that is caused by MERS-CoV, leading to severe disease with high mortality rates (approximately 35%) [1]. More recently, in 2019, a novel coronavirus (CoV) outbreak was reported in Wuhan, China [3]. This novel coronavirus disease (COVID-19), caused by severe acute respiratory syndrome coronavirus 2 (SARS-CoV-2), mainly attacks the respiratory system and causes acute respiratory distress syndrome (ARDS) which leads to medical disorder complications including death plus worldwide economic devastation [2]. Due to this exceptional outbreak, a number of pharmaceutical companies and academic researchers have been focused on developing and designing new anti-coronavirus candidate drugs to diminish this disease. So far, many anti-coronavirus vaccines have been developed which are effective, including those of Oxford/Astra-Zeneca, Janssen, Moderna, CoronaVac, and Covaxin. Despite these vaccines showing a high efficiency, it takes a long time to vaccinate all of the population worldwide, particularly in some developing countries. In addition, the vaccines may not meet all of the individuals’ needs due to medical complications [4,5,6]. The disadvantages, including unknown long-term side effects, occur rarely, but include severe allergic reactions such as anaphylaxis, short term immunization, and the need for booster doses. On similar lines, the trials to discover a new anti-coronavirus drug to reduce the transmission and inhibit viral infection are still ongoing for designed potential drug inhibitors. Due to the emergency pharmacological treatment for one of the most rapidly spread and easily transmissible coronaviruses in the world, SARS-CoV-2, drug repurposing was employed at the beginning of the global outbreak as one of the most common approaches to find a quick solution for the outbreak [6,7].

For example, the antiviral drugs suggested for pharmacological emergency use to block coronavirus (SARS-CoV-2) were umifenovir (arbidol), ruxolitinib, remdesivir, sofosbuvir, and chloroquine as an antimalarial drug [8]. The use of these drugs was accompanied by remarkable side effects. Nevertheless, remdesivir, an RNA-dependent RNA polymerase inhibitor and adenosine nucleotide analog that was initially developed to treat Ebola and Marburg viral infections, demonstrated promising in vitro and in vivo antiviral action against MERS-CoV and SARS-CoV-2 infections in experimental animal models, during clinical trials and in treatment protocols for COVID-19 patients [2].

Recently, another candidate suggested for COVID-19 treatment is a prodrug oral ribonucleoside analog with broad-spectrum anti-viral potential, namely Molnupiravir (EIDD-2801, MK-4482) [9]. The urgent development of a high efficacy and safe anti-viral drug to block or treat SARS-CoV-2 infection remains a challenge.

Due to the wide panel of the pharmacological activities and promising drug candidates for drug discovery, oxindole scaffolds and more specifically indole moieties as a class of heterocycles exhibited several pharmacological features including anti-viral, antimicrobial, anticancer, anti-inflammatory, antioxidant, analgesic, and antidiabetic potential [10,11]. Indeed, some merits of this type of privileged scaffold chemistry are the low cost, high chemical yield, eco-friendly synthesis, and simple workup procedure.

One of the virtual screening and *in silico* molecular docking studies suggested that the oxindole scaffold as a class of heterocyclic compound has the potential ability to bind with the main protease crystallized protein structure of COVID-19 (PDB ID: 6LU7) [12,13,14]. Out of the 30 oxindole derivatives computed, only four hits showed a strong binding affinity and low energy; interestingly, one hit of those leads was designed and synthesized by Barakat et al [15]. A recent study concluded about the potential scope of isatin derivatives for SARS-CoV-2 as protease inhibitors [14,15,16,17,18,19,20,21]. Therefore, to discover this spirooxindole scaffold as being anti-coronavirus is quite interesting.

**Figure 1:**
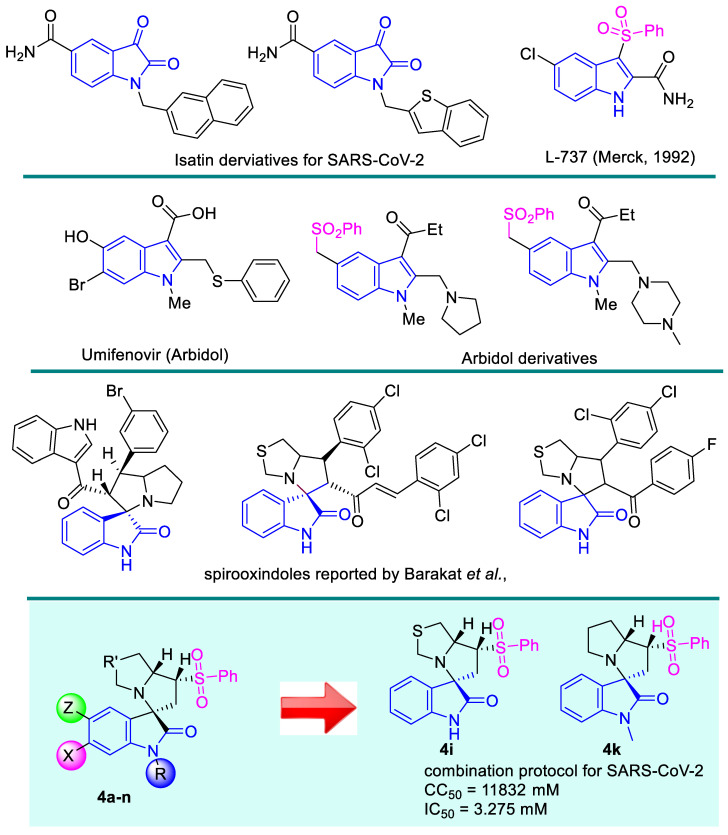
Representative examples of indole-derived moieties with pharmacological applications and our designed compounds as anti-viral drug for SARS-CoV-2.

Umifenovir (arbidol, Figure 1) which was developed in Russia as an indole containing an antiviral drug acts as a membrane fusion and an inhibitor for viral replication. Based on this finding, G. Sellitto et al used arbidol as a lead compound for structural derivatizations and reported the synthesis and evolution of new compounds having sulphone functionality. The arbidol derivatives exhibited strong antiviral activity against hepatitis C virus (HCV) with higher selectivity indices. The virus inhibition on entry and replication indicated that the phenylsulfonyl moiety played a crucial role for the anti-HCV activity [22]. Among the anti-viral agents designed, synthesized, and evaluated as protease inhibitors were non-peptidic heteroaromatic-based indole carboxylate small molecules having a phenylsulfone moiety and reported by Chiummiento et al. [23]. Other researchers have reported and stressed that indolylarylsulfones exhibited high potency as reverse transcriptase inhibitors which are a class of antiretroviral drugs for AIDS or HIV infection treatment [24,25,26,27,28,29,30,31,32,33].

To explore the phenylsulfone moiety in the spirooxindole framework and then to examine their activity against coronavirus is the source of a lot of attention for us. The goal of a combination protocol in a synergetic action is to combine medications that function through different or the same mechanisms, reducing the chances of side effects and developing resistance.

On the other hand, it is clinically recommended to combine two or more drugs as a cocktail protocol approach for the treatment of viral infections [34,35,36,37]. In addition, the use of an effective combinational protocol could reduce the effective concentration of compounds below the therapeutic plasma concentrations, providing better clinical benefits.

S. Yuan and H. Sun, as co-workers, demonstrated one representative example for an orally administered cocktail therapy for SARS-CoV-2 viral infection treatment. This preclinical cocktail therapy consisted of *N*-acetyl-*L*-cysteine combined with colloidal bismuth subsalicylate (BSS) or bismuth sub-citrate (CBS) assessed in vivo, and exhibited high efficacy against a wide range of coronavirus replications such as the recently circulating SARS-CoV-2 Alpha variant (B.1.1.7), the low pathogenic human coronavirus 229E (HCoV-229E), and the Middle East respiratory syndrome coronavirus (MERS-CoV), blocking or inhibiting several cell-based and viral-based targets including angiotensin-converting enzyme 2 (ACE2), helicase (Hel), main protease (Mpro), and papain-like protease (PLpro) [38].

Based on these findings and continuing with our research program for drug discoveries [39,40,41,42,43,44,45,46], here we report on the design, synthesis, and anti-viral evaluation of a new spirooxindole with a phenylsulfonyl moiety as a promising lead compound with high efficacy and a safer cocktail protocol to block and inhibit the outbreaks of a new coronavirus disease.

## 2. Results and Discussion

### 2.1. Synthesis of the Spirooxindole-Based Phenylsulfones ***4a–n***

**Scheme 1:**
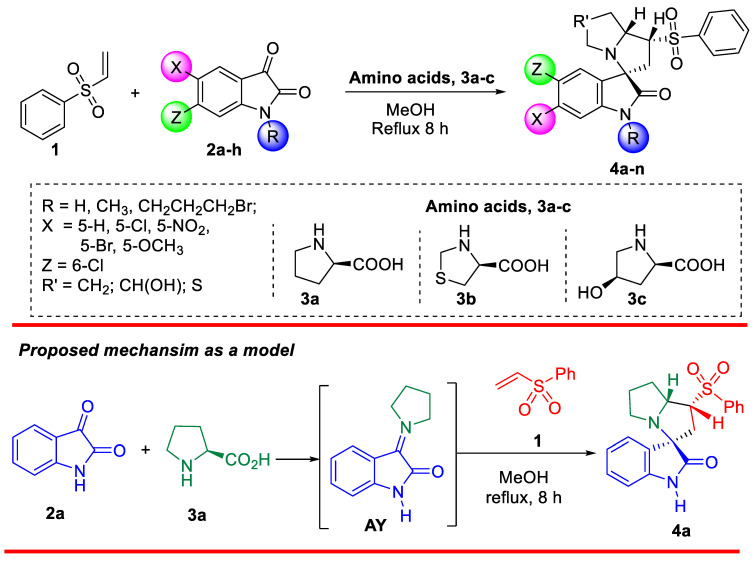
Synthesis of the spirooxindole-based phenylsulfone **4a–n.**

The design, construction, and synthesis of new materials with significant antiviral applications towards COVID-19 are a challenge. In this text we employed the one pot–multi component [3+2] cycloaddition (32CA) reaction approach for the synthesis of spirooxindole-based phenylsulfones as new materials [44]. The synthetic route is shown in Scheme 1. The spirooxindole-based phenylsulfone cycloadducts were obtained via 32CA reaction of phenyl vinyl sulfone **1** as the ethylene with the generated azomethine ylides (AYs) by reaction of many substituted isatins **2a–h** (Isatin **2a**, 5-Chloroisatin **2b**, 5-Nitroisatin **2c**, 5-Bromoisatin **2d,** 5-Methoxyisatin **2e,** 1-Methylindoline-2,3-dione **2f**, 1-(2-Bromoethyl)indoline-2,3-dione **2g**, 6-Chloroisatin **2h**) with three secondary amino acids **3a–c** (L-proline **3a**, L-thioproline **3b,** and (2*R*,4*R*)-4-hydroxypyrrolidine-2-carboxylic acid **3c**) under thermal conditions in MeOH for 8 h. The target spirooxindole-based phenylsulfone cycloadducts were afforded in a high chemical yield in a regioselective and diastereoselective fashion. Initially, the isatin reacted with l-proline to afford the azomethine ylide (AY), then reacted with the sulfone derivatives to afford the final product. The chemical architecture was assigned based on a number of spectrophotometric tools including ^1^HNMR, and ^13^CNMR spectral analysis. The stereochemical and regio-specific outcomes of the 32CA reactions were confirmed by HNMR and X-ray single diffraction analysis for the compound **4m** which has been published in our reported article [47,48].

### 2.2. Biological Studies

To test the preliminary antiviral activity of the synthesized spiro compounds against the highly pathogenic coronaviruses, SARS-CoV-2 “NRC-03-nhCoV” and MERS-CoV “NRCE-HKU270”, the half maximal cytotoxic “CC_50_” and virus-inhibitory (IC_50_) concentrations were determined using MTT assay and plaque reduction assay, respectively (Table 1). Except for the compounds **4b**, **4f**, **4g**, **4j**, and **4i–n**, the tested spiro-compounds showed high to moderate antiviral activity against SARS-CoV-2 ranging from 17 to 37 µM against NRC- 03-nhCoV and 15 to 74 µM against NRCE-HKU270 (Table 1). Interestingly, **4c**, **4i**, and **4k** showed the best selectivity index (SI > 10) against SARS-CoV-2 and MERS-CoV in VERO E6 cells (Table 1). These results compared with remdesivir as a drug control that the FDA approved as anti-SARS-CoV-2, but it had more cytotoxic effects and side effects on patients so there remains an urgent need to present alternatives against anti-SARS-CoV-2 with higher safety and lower side effects.

### 2.3. Combination Protocol

A drug combination protocol using anti-viral drugs provides several advantages as it will be more effective than monotherapy due to the synergistic effect of complementary drugs, have lower side effects, and lower toxicity due to reducing the doses. For these reasons, the use of combinations of drugs which may ultimately have a positive impact on alleviating COVID-19 severity have been studied [49,50]. Most drug combinations against COVID-19 have included the in vitro investigation of the recommended doses of FDA-approved drugs in 1:1 or 1:0.5 combinations [49,51]. For newly synthesized compounds, rare studies have considered investigating the synergistic or antagonistic effect of the compounds in combination against COVID-19.

**Figure 2:**
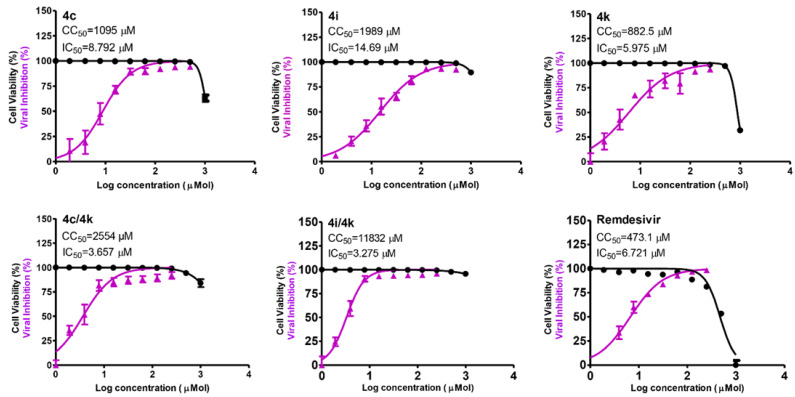
Half maximal cytotoxic concentration (CC_50_) in Vero-E6 cells and half maximal inhibitory concentration (IC_50_) of the tested compounds **4c, 4i,** and **4k**, individually and in combination, against NRC-03-nhCoV in Vero-E6 cells, compared with remdesivir as a drug control. The 50% inhibitory concentration (IC_50_) of each tested compound was calculated using nonlinear regression analysis in triplicate for each concentration used. The best fitting line was drawn between log concentrations and viral inhibition % using Graph Pad Prism software.

To further validate the anti-SARS-CoV-2 activity of the compounds **4c**, **4i**, and **4k**, colorimetric crystal violet assay was used as previously described [52]. After equal concentrations from each compound were prepared (10 mg/mL), the mixture was prepared with an equal volume from each compound (1:1). Accordingly, the compound **4k** showed the best IC_50_ values. Therefore, we included it in combination with the other two safe/active compounds (**4c** and **4i**). Interestingly, combinations with **4k** improved the IC_50_ of the compounds **4c** and **4i**. In addition, the mixture’s CC_50_ values improved when **4k** was used in combination with **4c**, and **4i** compared to each individual compound. Based on these results, the selectivity indices for the combinations **4c/4k** and **4i/4k** were remarkably improved, to be 698.4 and 3612.8, respectively (Figure 2).

Taking into consideration that remdesivir is one of the most promising anti-COVID-19 drugs, as demonstrated by previous in vitro and clinical studies [2,53,54], the inhibitory concentrations for the selected combinations **4c/4k** and **4i/4k** were lower than that for the control remdesivir drug (IC_50_ = 6.721 µM). Interestingly, the IC_50_ values for the selected combinations were also potent compared to the other FDA-approved drugs that are applied in COVID-19 treatment protocols including viral protease inhibitors Lopinavir (IC_50_ = 26.63 µM) and Ritonavir (IC_50_ ≥100 µM), RdRp inhibitors such as Favipiravir (IC_50_ = 61.88 µM) and Ribavirin (IC_50_ = 70 µM), and other small-molecule inhibitors including Azithromycin (IC_50_ = 2.12 µM), and Hydroxychloroquine (IC_50_ = 4.51 µM) [53].

### 2.4. Molecular Docking Study

#### 2.4.1. Docking against SARSCoV-2 against RNA Polymerase (PDB:ID: 6m71)

**Figure 3:**
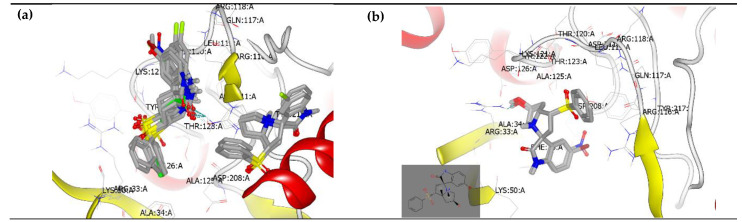
Visual representation for compounds docked against (PDBID: 6m71) visualized by vid application (**a**) shape alignment for synthesized compounds **4i, 4n, 4b, 4e, 4m, 4f,** and **4j** (left domain of receptor). Compounds **4a, 4g** and **4k** (right domain of receptor); (**b**) Compounds **4c**, and **4d** have the same binding mode and pose with formation of HB with Arg: 33A.

The compounds exhibited different binding modes and poses against the target protein. According to the binding mode and site of interaction with the receptor, all compounds are classified in to three categories:

1. Compounds **4n**, **4b**, **4e**, **4m**, **4f**, **4j**, and **4i** have the same binding mode and these compounds were originated from secondary amino acids L-proline **3a**, and L-thioproline **3b** and were docked with complete overlay and with the detection of hydrogen bond (HB) with Arg: 116A through the carbonyl of oxoindoline moiety, Figure 3a (left domain of receptor);
2. Compounds **4a**, **4g**, and **4k** were prepared from secondary amino acid L-proline **3a**. These compounds connected with same amino acids cleft with complete overlay and similarity without detection of any hydrogen bonds (HBs), Figure 3a (right domain of receptor);
3. Compounds **4d**, **4l, 4h**, and **4c** exhibited the same binding mode and pose with formation of HB with Arg: 33A through the hydroxyl functionality of pyrrolidine ring, Figure 3b. These compounds were synthesized from the secondary amino acids L-proline **3a** and 2*R*,4*R*)-4-hydroxypyrrolidine-2-carboxylic acid (**3c).** Compound **4h** formed HB with Thr:120A through NH of oxoindoline moiety, Figure S28. Compound **4l** illustrated specific binding pose through hydrophobic–hydrophobic interaction, Figure S29.

**Figure 4:**
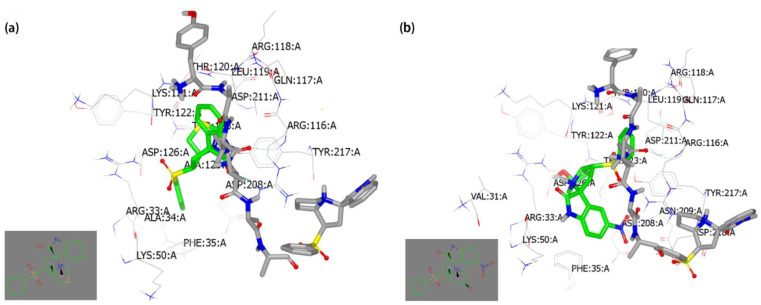
Visual representation for the combination compounds docked against (PDBID: 6m71) (**a**) snapshot of both compounds **4k** (grey color) and **4i** (green color) in presence of co-crystalized standard ligand (grey). The HB illustrated in green color; (**b**) snapshot of both compounds **4k** (grey color) and **4c** (green color) in presence of co-crystalized standard ligand (grey).

The combination study was designed between compound **4k** (second category) with compound **4i** (first category); and compound **4k** (second category) with compound **4c** (third category). Interestingly, combinations with **4k** improved the IC_50_ of the compounds **4c** and **4i**. In addition, the mixture’s CC_50_ values were improved. From Figure 4a, compound **4k** (grey color) binds with the amino acid cleft which differs from those interacting with compound **4i** (green color). Compound **4i** located in the site of the receptor close to the binding region of co-crystalized ligand. They formed HB with Arg: 116A and His:99A, respectively. Concerning the combination (compound **4k** and **4c**), compound **4c** (green color) adopted the same region as shown before from compound **4i** but in a different binding mode and pose, Figure 4b. Compound **4c** formed weak HB with Arg: 33A through its hydroxyl functionality of the pyrrolidine ring, Figure 4b 55.

#### 2.4.2. Docking Study with MERS-CoV Viral Proteins nsp5 (PDB:ID: 4ylu)

**Figure 5:**
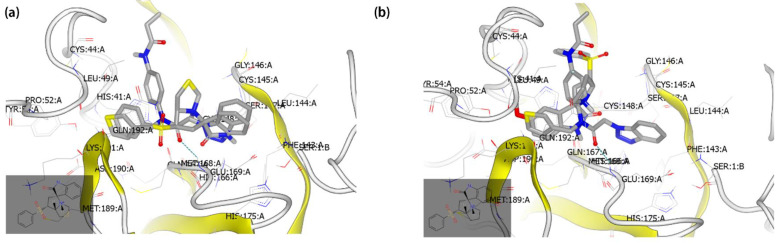
Visual representation for compounds docked against (PDBID: 4ylu) visualized by vida application: (**a**) compound **4i** docked with the receptor through hydrophobic–hydrophobic interaction; (**b**) compound **4h** docked in a different domain.

In order to examine the activity of these compounds against the MERS-CoV virus, the docking protocol was employed here against the main protease of MERS-CoV (PDB ID: 4ylu [56]). Both compound **4h** and **4i** were the most potent derivatives. Compound **4i** docked with the receptor with hydrophobic–hydrophobic interaction in the same amino acids’ clefts interacted with the co-crystalized ligand, Figure 5a, however compound **4h** participated in a docking pose in a different domain, Figure 5b.

### 2.5. ADMET Analysis

**Figure 6:**
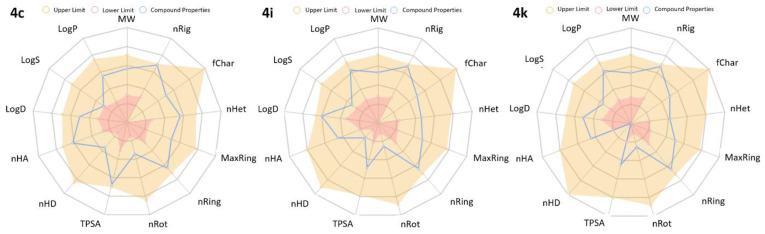
*In silico* ADMET properties of selected spirooxindole analogs predicted by ADMETlab.

Due to adverse pharmacokinetic properties, many of the prospective drug candidates never reach clinical trials. To examine the fundamental pharmacokinetic features of a compound, *in silico* ADMET analysis provides a valid alternative to earlier stage experiments to increase the success rate of clinical development. The bioavailability, pharmacokinetics, and toxicity of **4c**, **4i**, and **4k** were attained by using ADMETlab and the obtained results are summarized in Table 2. The bioavailability and physiochemical properties were evaluated by plotting a radar representing 13 properties (Figure 6). It is interesting to note that all of the properties were within their optimal ranges, which showed that **4c**, **4i**, and **4k** had good oral bioavailability and druggability. Similarly, all of the compounds were predicted to follow the Lipinski rule of five with high gastrointestinal absorption. To further evaluate the toxicity and cross-reactivities of **4c**, **4i**, and **4k**, the acute toxicity and PAINS were predicted. All of the three compounds were found to be non-toxic and non-cross-reactive. Taken together, **4c**, **4i**, and **4k** were in significant agreement with the given criteria to be considered as drug-like.

### 2.6. Molecular Dynamics Simulation

**Figure 7:**
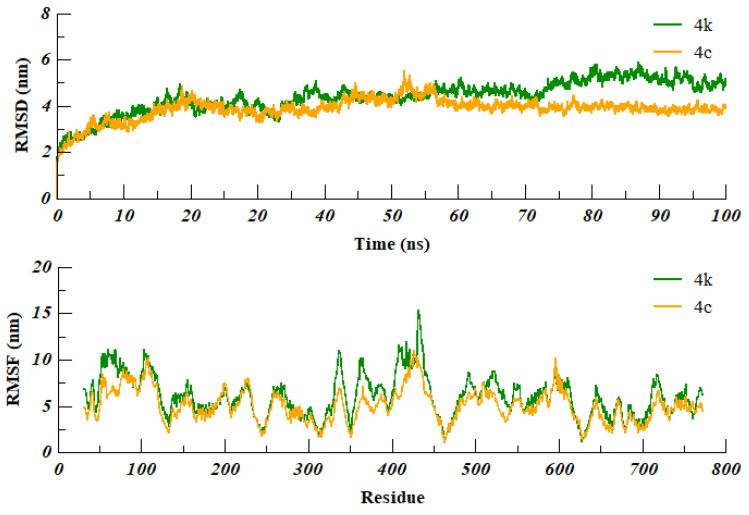
RMSD and RMSF plot of **4c** and **4k** in complex with RdRp of SARS-CoV-2 over the 100ns of simulation.

The compounds **4c** and **4k** with the best selectivity index and significant antiviral activity against SARS-CoV-2 were subjected to molecular dynamics (MD) simulation to evaluate the time-dependent dynamics and stability of protein–ligand complexes. The docked pose of **4c** and **4k** in complex with RdRp of SARS-CoV-2 were subjected to 100ns of simulation and the Root Mean Square Deviation (RMSD) and Root Mean Square Fluctuation (RMSF) were calculated (Figure 7). The RMSD plot of **4k**-RdRp showed variable fluctuations throughout the 100ns of simulation with the average RMSD of 4.3 ± 0.70 nm. The **4c**-RdRp projected the more stable RMSD in comparison with the **4k** complex with an average RMSD of 3.8 ± 0.47 nm. The **4c**-RdRp complex was converged after 55ns and remained stable till the end of the simulation. To further evaluate the ligand-induced flexibility of SARS-CoV-2 RdRp residues, RMSF was calculated. Consistent with the RMSD results, the RSMF plot of the **4c** complex showed a lesser magnitude of fluctuations as compared to the **4k** complex. The average RMSF for the **4k**-RdRp and **4c**-RdRp complexes was found to be 6.04 ± 2.23 nm and 5.05 ± 1.78 nm, respectively. By analyzing the results, it was inferred that the experimental and theoretical results were consistent with one another.

## 3. Methodology/Experimental Section

### 3.1. General

The ^1^H-NMR and ^13^C-NMR spectra of **4a–n** were recorded on a JEOL 400-MHz spectrometer (JEOL, Ltd, Tokyo, Japan) at ambient temperature. DMSO-*d*_6_ was used as the solvent; the chemical shifts (*δ*) are given in ppm.

Synthesis of the Spirooxindole-Based Phenylsulfone **4a–n (GP1)**

A mixture of phenyl vinyl sulfone **1** (0.5 mmol), isatin derivatives **2a–h** (0.5 mmol), and amino acids **3a–c** (0.5 mmol) in methanol (10 mL) was refluxed in an oil bath for the appropriate time of 8 h. After completion of the reaction, as evident from TLC, the reaction was maintained at room temperature overnight, and the solid crystalline product was filtered off without any further purification.

#### 3.1.1. (1’*R*,3*R*,7a’*R*)-1’-(Phenylsulfonyl)-1’,2’,5’,6’,7’,7a’-hexahydrospiro[indoline-3,3’-pyrrolizin]-2-one **4a**

**1** (84 mg, 0.5 mmol), isatin **2a** (73.5 mg, 0.5 mmol) and L-proline **3a** (57.5 mg, 0.5 mmol) were reacted according to GP1 for 8 h and yielded white solid phenylsulfone spirooxindole **4a** (166 mg, 90%); m.p.298 °C; ^1^H NMR (400 MHz, DMSO-*d*_6_) *δ* 10.39 (s, 1H), 8.00 (d, *J* = 7.8 Hz, 2H), 7.82 (t, *J* = 7.4 Hz, 1H), 7.71 (d, *J* = 7.6 Hz, 2H), 7.36–7.24 (m, 2H), 7.05 (t, *J* = 7.6 Hz, 1H), 6.83 (d, *J* = 7.9 Hz, 1H), 4.21 (d, *J* = 8.9 Hz, 2H), 4.02 (d, *J* = 8.9 Hz, 2H), 3.94–3.77 (m, 2H), 2.81 (dd, *J* = 10.3, 6.5 Hz, 2H), 2.27 (dd, *J* = 13.3, 6.8 Hz, 1H); ^13^C NMR (101 MHz, DMSO-*d*_6_) δ 179.48, 172.96, 143.19, 139.19, 134.99, 130.29, 128.41, 126.09, 125.84, 122.38, 69.56, 68.82, 65.52, 54.51, 53.15, 36.61, 31.41, 22.52, 14.42; [Anal. Calcd. for C_20_H_20_N_2_O_3_S: C, 65.20; H, 5.47; N, 7.60; Found: C, 65.29; H, 5.58; N, 7.65]; LC/MS (ESI, *m/z*): found 369.16 [M+H]^+^; Exact Mass: 368.12.

#### 3.1.2. (3*R*,7’*R*,7a’*R*)-5-Chloro-7’-(phenylsulfonyl)-1’,6’,7’,7a’-tetrahydro-3’*H*-spiro[indoline-3,5’-pyrrolo [1,2-*c*]thiazole]-2-one **4b**

**1** (84 mg, 0.5 mmol), 5-Cl-isatin **2b** (90.5 mg, 0.5 mmol) and L- thioproline **3b** (66.5 mg, 0.5 mmol) were reacted according to GP1 for 8 h and yielded white solid phenylsulfone spirooxindole **4b** (194 mg, 92%); m.p.282 °C; ^1^H NMR (400 MHz, DMSO-*D*_6_) δ 10.53 (s, 1H), 8.01 (d, *J* = 7.9 Hz, 2H), 7.82 (t, *J* = 7.4 Hz, 1H), 7.71 (t, *J* = 7.7 Hz, 2H), 7.40–7.31 (m, 2H), 6.84 (d, *J* = 8.1 Hz, 1H), 4.61 (dt, *J* = 12.2, 6.5 Hz, 1H), 3.91 (d, *J* = 10.0 Hz, 1H), 3.80 (dt, *J* = 9.5, 6.2 Hz, 1H), 3.35 (d, *J* = 17.4 Hz, 5H), 3.20–3.00 (m, 2H), 2.57 (d, *J* = 13.2 Hz, 1H), 2.29 (dd, *J* = 13.3, 6.8 Hz, 1H); ^13^C NMR (101 MHz, DMSO-*d*_6_) δ 179.55, 144.93, 139.29, 135.14, 134.96, 130.44, 128.52, 127.64, 125.12, 122.17, 110.52, 69.35, 68.96, 62.52, 53.28, 34.56, 32.64; [Anal. Calcd. for C_19_H_17_ClN_2_O_3_S_2_: C, 54.22; H, 4.07; Cl, 8.42; N, 6.66; Found: C, 54.32; H, 4.11; N, 6.77]; LC/MS (ESI, *m/z*): found 421.12 [M+H]^+^; Exact Mass: 420.04.

#### 3.1.3. (1’*R*,3*R*,6’*S*,7a’*R*)-6’-Hydroxy-5-nitro-1’-(phenylsulfonyl)-1’,2’,5’,6’,7’,7a’-hexahydrospiro[indoline-3,3’-pyrrolizin]-2-one **4c**

**1** (84 mg, 0.5 mmol), 5-NO_2_-isatin **2c** (96mg, 0.5 mmol) and (2R,4R)-4-hydroxypyrrolidine-2-carboxylic acid **3c** (66.5 mg, 0.5 mmol) were reacted according to GP1 for 8 h and yielded yellow solid phenylsulfone spirooxindole **4c** (185 mg, 86%); m.p.141 °C^1^H NMR (400 MHz, DMSO-*D*_6_) δ 11.16 (s, 1H), 7.97 (d, *J* = 7.5 Hz, 1H), 7.82 (d, *J* = 2.2 Hz, 1H), 7.76–7.66 (m, 2H), 7.56 (d, *J* = 7.8 Hz, 2H), 7.06–6.98 (m, 2H), 4.79 (s, 2H), 4.35 (dd, *J* = 13.0, 6.1 Hz, 1H), 4.27 (d, *J* = 6.2 Hz, 1H), 4.19 (d, *J* = 7.6 Hz, 1H), 4.09–3.96 (m, 1H), 2.31 – 2.16 (m, 3H), 1.95–1.85 (m, 1H), 1.61 (ddd, *J* = 13.3, 9.0, 5.0 Hz, 1H); ^13^C NMR (101 MHz, DMSO-*d*_6_) δ 179.08, 178.50, 149.91, 142.73, 142.13, 140.50, 138.69, 138.50, 129.98, 124.90, 105.12, 70.34, 70.19, 68.71, 64.85, 57.59, 31.13, 23.63; [Anal. Calcd. for C_20_H_19_N_3_O_6_S: C, 55.94; H, 4.46; N, 9.78; Found: C, 55.90; H, 4.40; N, 9.70]; LC/MS (ESI, *m/z*): found 430.18 [M+H]^+^; Exact Mass: 429.10.

#### 3.1.4. (1’*R*,3*R*,6’*S*,7a’*R*)-5-Bromo-6’-hydroxy-1’-(phenylsulfonyl)-1’,2’,5’,6’,7’,7a’-hexahydrospiro[indoline-3,3’-pyrrolizin]-2-one **4d**

**1** (84 mg, 0.5 mmol), 5-Br-isatin **2d** (113 mg, 0.5 mmol) and (2R,4R)-4-hydroxypyrrolidine-2-carboxylic acid **3c** (66.5 mg, 0.5 mmol) were reacted according to GP1 for 8 h and yielded faint brown solid phenylsulfone spirooxindole **4d** (185 mg, 84%); m.p.220 °C; ^1^H NMR (400 MHz, DMSO-*d*_6_) δ 10.55 (s, 1H), 8.01 (d, *J* = 7.7 Hz, 2H), 7.81 (q, *J* = 6.9, 6.4 Hz, 1H), 7.70 (p, *J* = 7.5 Hz, 2H), 7.48 (d, *J* = 7.3 Hz, 2H), 6.79 (d, *J* = 8.6 Hz, 1H), 4.60 (dt, *J* = 11.9, 6.2 Hz, 1H), 4.21 (d, *J* = 8.9 Hz, 1H), 4.02 (d, *J* = 8.8 Hz, 1H), 3.91 (d, *J* = 10.2 Hz, 1H), 3.87–3.74 (m, 2H), 3.36 (d, *J* = 9.8 Hz, 1H), 3.18–2.99 (m, 3H), 2.81 (dd, *J* = 10.2, 6.6 Hz, 1H), 2.56 (d, *J* = 12.9 Hz, 1H), 2.28 (dd, *J* = 13.3, 7.0 Hz, 1H); ^13^C NMR (101 MHz, DMSO- *d*_6_) δ 179.15, 173.08, 142.72, 139.28, 130.62, 128.61, 114.17, 69.76, 68.93, 65.64, 54.62, 53.36, 36.75, 34.68; [Anal. Calcd. for C_20_H_19_BrN_2_O_4_S: C, 51.84; H, 4.13; N, 6.05; Found: C, 51.91; H, 4.18; N, 6.09]; LC/MS (ESI, *m/z*): found 462.18 [M+H]^+^; Exact Mass: 462.02.

#### 3.1.5. (3*R*,7’*R*,7a’*R*)-5-Nitro-7’-(phenylsulfonyl)-1’,6’,7’,7a’-tetrahydro-3’H-spiro[indoline-3,5’-pyrrolo [1,2-*c*]thiazole]-2-one **4e**

**1** (84 mg, 0.5 mmol), 5-NO_2_-isatin **2c** (96 mg, 0.5 mmol) and L- thioproline **3b** (66.5 mg, 0.5 mmol) were reacted according to GP1 for 8 h and yielded yellow solid phenylsulfone spirooxindole **4e** (173 mg, 80%); m.p.95 °C; ^1^H NMR (400 MHz, CDCl_3_) δ 8.99 (s, 1H), 8.28 (dd, *J* = 8.8, 2.2 Hz, 1H), 7.64 (dd, *J* = 8.0, 5.8 Hz, 5H), 7.50 (d, *J* = 7.6 Hz, 2H), 7.01 (t, *J* = 8.0 Hz, 2H), 3.83 (d, *J* = 11.5 Hz, 2H), 3.80 (s, 1H), 3.19 (dd, *J* = 11.9, 6.9 Hz, 2H), 3.03 (dd, *J* = 11.7, 2.7 Hz, 1H); ^13^C NMR (101 MHz, CDCl_3_) δ 178.45, 147.73, 142.67, 138.52, 138.24, 134.62, 129.56, 129.51, 128.40, 128.05, 128.00, 127.52, 126.15, 110.38, 71.91, 71.04, 67.53, 54.16, 38.14; [Anal. Calcd. for C_19_H_17_N_3_O_5_S_2_: C, 52.89; H, 3.97; N, 9.74; Found: C, 52.98; H, 3.98; N, 9.85]; LC/MS (ESI, *m/z*): found 432.12 [M+H]^+^; Exact Mass: 431.06.

#### 3.1.6. (3*R*,7’*R*,7a’*R*)-5-Methoxy-7’-(phenylsulfonyl)-1’,6’,7’,7a’-tetrahydro-3’*H*-spiro[indoline-3,5’-pyrrolo [1,2-*c*]thiazole]-2-one **4f**

**1** (84 mg, 0.5 mmol), 5-MeO-isatin **2e** (88.5 mg, 0.5 mmol) and L- thioproline **3b** (66.5 mg, 0.5 mmol) were reacted according to GP1 for 8 h and yielded yellow solid phenylsulfone spirooxindole **4f** (171 mg, 82%); m.p.105 °C; ^1^H NMR (400 MHz, DMSO-*d*_6_) δ 10.21 (s, 1H), 8.01 (d, *J* = 7.8 Hz, 2H), 7.82 (t, *J* = 7.3 Hz, 1H), 7.71 (t, *J* = 7.7 Hz, 2H), 6.98 (d, *J* = 2.7 Hz, 1H), 6.87 (dd, *J* = 8.1, 2.8 Hz, 1H), 6.74 (d, *J* = 8.6 Hz, 1H), 4.62 (dt, *J* = 12.4, 6.5 Hz, 1H), 3.91 (d, *J* = 9.7 Hz, 1H), 3.82–3.71 (m, 1H), 3.75 (s, 2H), 3.38 (d, *J* = 9.7 Hz, 2H), 3.33 (s, 2H), 3.16–3.00 (m, 2H), 2.28–2.20 (m, 1H); ^13^C NMR (101 MHz, DMSO-*d*_6_) δ 179.58, 155.45, 139.34, 136.38, 130.39, 127.31, 119.34, 113.81, 112.62, 70.14, 58.53, 34.69, 12.34; [Anal. Calcd. for C_20_H_20_N_2_O_4_S_2_: C, 57.67; H, 4.84; N, 6.73; Found: C, 57.75; H, 4.94; N, 6.86]; LC/MS (ESI, *m/z*): found 417.17 [M+H]^+^; Exact Mass: 416.09.

#### 3.1.7. (1’*R*,3*R*,7a’*R*)-5-Chloro-1’-(phenylsulfonyl)-1’,2’,5’,6’,7’,7a’-hexahydrospiro[indoline-3,3’-pyrrolizin]-2-one **4g**

**1** (84 mg, 0.5 mmol), 5-Cl-isatin **2b** (90.5 mg, 0.5 mmol) and L- proline **3a** (57.5 mg, 0.5 mmol) were reacted according to GP1 for 8 h and yielded yellow solid phenylsulfone spirooxindole **4g** (177 mg, 88%); m.p. 254 °C; ^1^H NMR (400 MHz, DMSO-*D*_6_) δ 10.44 (s, 1H), 7.95 (d, *J* = 7.9 Hz, 2H), 7.80–7.71 (m, 1H), 7.66 (t, *J* = 7.7 Hz, 2H), 7.53 (s, 1H), 7.34–7.27 (m, 1H), 6.80 (d, *J* = 8.6 Hz, 1H), 4.47 (dt, *J* = 10.8, 7.7 Hz, 1H), 4.04 (q, *J* = 8.2 Hz, 1H), 2.83 (td, *J* = 9.2, 6.4 Hz, 1H), 2.58 (t, *J* = 11.8 Hz, 1H), 2.50 (s, 3H), 2.47 (d, *J* = 7.6 Hz, 0H), 2.34 (t, *J* = 10.9 Hz, 1H), 2.03 (dd, *J* = 13.1, 7.3 Hz, 1H), 1.92 (dt, *J* = 13.9, 7.4 Hz, 2H), 1.64 (q, *J* = 9.2, 8.3 Hz, 1H); ^13^C NMR (101 MHz, DMSO-*D*_6_) δ 179.37, 142.26, 140.72, 130.07, 129.25, 128.96, 128.42, 126.19, 92.09, 78.91, 69.36, 66.27, 61.69, 52.61, 27.44; [Anal. Calcd. for C_20_H_19_ClN_2_O_3_S: C, 59.62; H, 4.75; N, 6.95; Found: C, 59.58; H, 4.73; N, 6.90]; LC/MS (ESI, *m/z*): found 403.13 [M+H]^+^; Exact Mass: 402.08.

#### 3.1.8. (1’*R*,3*R*,7a’*R*)-5-Methoxy-1’-(phenylsulfonyl)-1’,2’,5’,6’,7’,7a’-hexahydrospiro[indoline-3,3’-pyrrolizin]-2-one **4h**

**1** (84 mg, 0.5 mmol), 5-MeO-isatin **2e** (88.5 mg, 0.5 mmol) and L-proline **3b** (57.5 mg, 0.5 mmol) were reacted according to GP1 for 8 h and yielded faint brown solid phenylsulfone spirooxindole **4h** (172 mg, 86%); m.p.240 °C; ^1^H NMR (400 MHz, DMSO-*d*_6_) δ 10.13 (s, 1H), 7.94 (d, *J* = 7.8 Hz, 2H), 7.76 (t, *J* = 7.3 Hz, 1H), 7.66 (t, *J* = 7.6 Hz, 2H), 7.03 (d, *J* = 2.7 Hz, 1H), 6.83 (dd, *J* = 8.7, 2.7 Hz, 1H), 6.71 (d, *J* = 8.6 Hz, 1H), 4.49 (dt, *J* = 11.0, 7.9 Hz, 1H), 4.02 (q, *J* = 8.2 Hz, 1H), 3.74 (s, 2H), 2.86 (td, *J* = 9.2, 6.2 Hz, 1H), 2.61–2.52 (m, 1H), 2.40–2.24 (m, 1H), 1.93 (ddt, *J* = 20.2, 14.4, 7.4 Hz, 3H), 1.69–1.56 (m, 1H); ^13^C NMR (101 MHz, DMSO-*d*_6_) δ 179.63, 155.26, 140.76, 136.49, 133.55, 131.02, 130.21, 128.37, 128.33, 116.18, 112.47, 69.73, 64.69, 57.35, 48.66, 32.48, 27.74; [Anal. Calcd. for C_21_H_22_N_2_O_4_S: C, 63.30; H, 5.57; N, 7.03; Found: C, 63.38; H, 5.69; N, 7.11]; LC/MS (ESI, *m/z*): found 399.19 [M+H]^+^; Exact Mass: 398.13.

#### 3.1.9. (3*R*,7’*R*,7a’*R*)-7’-(Phenylsulfonyl)-1’,6’,7’,7a’-tetrahydro-3’*H*-spiro[indoline-3,5’-pyrrolo [1,2-*c*]thiazole]-2-one **4i**

**1** (84 mg, 0.5 mmol), isatin **2a** (73.5 mg, 0.5 mmol) and L- thioproline **3b** (66.5 mg, 0.5 mmol) were reacted according to GP1 for 8 h and yielded white solid phenylsulfone spirooxindole **4i** (168 mg, 87%); m.p. 260 °C; ^1^H NMR (400 MHz, DMSO-*d*_6_) δ 10.31 (s, 1H), 7.94 (d, *J* = 7.9 Hz, 2H), 7.76 (t, *J* = 7.3 Hz, 1H), 7.66 (t, *J* = 7.5 Hz, 2H), 7.40 (d, *J* = 7.4 Hz, 1H), 7.25 (t, *J* = 7.7 Hz, 1H), 7.00 (t, *J* = 7.4 Hz, 1H), 6.80 (d, *J* = 7.9 Hz, 1H), 4.48 (dt, *J* = 10.9, 7.6 Hz, 1H), 4.03 (q, *J* = 8.0 Hz, 1H), 2.83 (td, *J* = 9.4, 6.5 Hz, 1H), 2.31 (ddd, *J* = 20.3, 11.8, 8.7 Hz, 1H), 1.94 (ddt, *J* = 34.9, 14.9, 7.7 Hz, 3H), 1.69–1.56 (m, 1H); ^13^C NMR (101 MHz, DMSO-*d*_6_) δ 179.73, 143.30, 140.71, 134.97, 130.12, 128.18, 127.16, 120.36, 109.47, 69.40, 64.70, 50.64, 26.60; [Anal. Calcd. for C_19_H_18_N_2_O_3_S_2_: C, 59.05; H, 4.69; N, 7.25; Found: C, 59.15; H, 4.77; N, 7.36]; LC/MS (ESI, *m/z*): found 387.17 [M+H]^+^; Exact Mass: 386.08.

#### 3.1.10. (1’*R*,3*R*,7a’*R*)-5-Bromo-1’-(phenylsulfonyl)-1’,2’,5’,6’,7’,7a’-hexahydrospiro[indoline-3,3’-pyrrolizin]-2-one **4j**

**1** (84 mg, 0.5 mmol), 5-Br-isatin **2d** (113 mg, 0.5 mmol) and L-proline **3a** (57.5 mg, 0.5 mmol) were reacted according to GP1 for 8 h and yielded faint grey solid phenylsulfone spirooxindole **4j** (199 mg, 89%); m.p.250 °C; ^1^H NMR (400 MHz, DMSO-*d*_6_) δ 10.45 (s, 1H), 7.95 (d, *J* = 7.6 Hz, 2H), 7.76 (t, *J* = 7.4 Hz, 1H), 7.70–7.61 (m, 3H), 7.44 (d, *J* = 8.2 Hz, 1H), 6.76 (d, *J* = 8.3 Hz, 1H), 4.45 (t, *J* = 9.2 Hz, 1H), 4.03 (q, *J* = 8.1 Hz, 1H), 2.83 (q, *J* = 8.2, 7.7 Hz, 1H), 2.59 (t, *J* = 12.0 Hz, 1H), 2.46 (d, *J* = 9.5 Hz, 1H), 2.32 (q, *J* = 10.4, 9.8 Hz, 1H), 2.04 (dd, *J* = 13.1, 7.3 Hz, 1H), 1.92 (dt, *J* = 13.6, 7.3 Hz, 2H), 1.64 (q, *J* = 9.9 Hz, 1H); ^13^C NMR (101 MHz, DMSO-*d*_6_) δ 179.25, 142.68, 140.73, 134.58, 133.00, 132.60, 130.23, 129.66, 128.30, 113.82, 110.26, 108.28, 100.23, 69.30, 64.72, 61.14, 49.00, 33.84, 26.62; [Anal. Calcd. For C_20_H_19_BrN_2_O_3_S: C, 53.70; H, 4.28; N, 6.26; Found: C, 53.78; H, 4.33; N, 6.34]; LC/MS (ESI, *m/z*): found 447.15 [M+H]^+^; Exact Mass: 446.03.

#### 3.1.11. (1’*R*,3*R*,7a’*R*)-1-Methyl-1’-(phenylsulfonyl)-1’,2’,5’,6’,7’,7a’-hexahydrospiro[indoline-3,3’-pyrrolizin]-2-one **4k**

**1** (84 mg, 0.5 mmol), N-Me-isatin **2f** (80.5 mg, 0.5 mmol) and L- proline **3a** (57.5 mg, 0.5 mmol) were reacted according to GP1 for 8 h and yielded yellow solid phenylsulfone spirooxindole **4k** (153 mg, 80%); m.p.71 °C; ^1^H NMR (400 MHz, DMSO-*d*_6_) δ 7.95 (s, 1H), 7.65 (s, 1H), 7.49 (dd, *J* = 19.6, 7.7 Hz, 2H), 7.35 (dq, *J* = 17.2, 8.6, 7.9 Hz, 2H), 7.08 (t, *J* = 7.5 Hz, 1H), 7.01 (dd, *J* = 26.7, 7.7 Hz, 1H), 4.51 (dt, *J* = 11.5, 7.8 Hz, 1H), 4.06 (q, *J* = 8.1 Hz, 1H), 3.32 (s, 1H), 3.00 (s, 2H), 2.85 (td, *J* = 9.3, 6.2 Hz, 1H), 2.80 (s, 1H), 2.55 (d, *J* = 12.3 Hz, 1H), 2.47 (t, *J* = 7.8 Hz, 1H), 2.39–2.26 (m, 1H), 2.04–1.87 (m, 3H), 1.72–1.48 (m, 2H); ^13^C NMR (101 MHz, DMSO-*d*_6_) δ 177.72, 144.79, 140.73, 138.53, 135.92, 130.39, 129.16, 128.41, 126.41, 123.10, 110.65, 108.12, 71.01, 69.15, 63.90, 62.48, 29.32.; [Anal. Calcd. for C_21_H_22_N_2_O_3_S: C, 65.95; H, 5.80; N, 7.32; Found: C, 66.01; H, 5.85; N, 7.40]; LC/MS (ESI, *m/z*): found 383.28 [M+H]^+^; Exact Mass: 382.14.

#### 3.1.12. (1’*R*,3*R*,7a’*R*)-1-(2-Bromoethyl)-1’-(phenylsulfonyl)-1’,2’,5’,6’,7’,7a’-hexahydrospiro[indoline-3,3’-pyrrolizin]-2-one **4l**

**1** (84 mg, 0.5 mmol), *N*-Br-Et-isatin **2g** (126.5 mg, 0.5 mmol) and L- proline **3a** (57.5 mg, 0.5 mmol) were reacted according to GP1 for 8 h and yielded yellow solid phenylsulfone spirooxindole **4l** (216 mg, 91%); m.p.70 °C; ^1^H NMR (400 MHz, DMSO-*d*_6_) δ 7.96 (d, *J* = 7.9 Hz, 1H), 7.81–7.62 (m, 3H), 7.52 (t, *J* = 7.7 Hz, 2H), 7.35 (h, *J* = 6.6, 5.6 Hz, 3H), 7.09 (dq, *J* = 24.3, 7.6 Hz, 3H), 4.33 (dd, *J* = 13.1, 6.0 Hz, 1H), 3.99 (q, *J* = 7.2 Hz, 1H), 3.83 (s, 0H), 3.66 (ddt, *J* = 26.4, 19.9, 6.1 Hz, 3H), 2.39–2.25 (m, 1H), 2.25 – 2.06 (m, 1H), 1.96 (qd, *J* = 10.9, 10.4, 4.8 Hz, 2H), 1.85–1.68 (m, 1H), 1.65 (d, *J* = 8.2 Hz, 1H), 1.56 (p, *J* = 8.5 Hz, 1H); ^13^C NMR (101 MHz, DMSO-*d*_6_) δ 143.41, 138.53, 134.72, 130.28, 129.78, 129.36, 129.06, 128.51, 128.36, 127.97, 126.89, 123.31, 122.23, 109.74, 97.23, 72.43, 70.14, 66.67, 65.09, 32.50, 32.30, 29.71, 28.60, 26.59; [Anal. Calcd. for C_22_H_23_BrN_2_O_3_S: C, 55.58; H, 4.88; N, 5.89; Found: C, 55.64; H, 4.93; N, 5.97]; LC/MS (ESI, *m/z*): found 475.09 [M+H]^+^; Exact Mass: 474.06.

#### 3.1.13. ((1’*R*,3*R*,7a’*R*)-6-Chloro-1’-(phenylsulfonyl)-1’,2’,5’,6’,7’,7a’-hexahydrospiro[indoline-3,3’-pyrrolizin]-2-one **4m**

The spectral data are matched with the reported literature [47,48].

#### 3.1.14. (3*R*,7’*R*,7a’*R*)-6-Chloro-7’-(phenylsulfonyl)-1’,6’,7’,7a’-tetrahydro-3’*H*-spiro[indoline-3,5’-pyrrolo [1,2-*c*]thiazole]-2-one **4n**

**1** (84 mg, 0.5 mmol), 6-Cl-isatin **2h** (90.5 mg, 0.5 mmol) and L- thioproline **3b** (66.5 mg, 0.5 mmol) were reacted according to GP1 for 8 h and yielded white solid phenylsulfone spirooxindole **4n** (189 mg, 90%); m.p.271 °C; ^1^H NMR (400 MHz, DMSO-*d*_6_) δ 10.54 (s, 1H), 8.00 (d, *J* = 7.7 Hz, 2H), 7.82 (t, *J* = 7.5 Hz, 1H), 7.70 (t, *J* = 7.8 Hz, 2H), 7.35 (d, *J* = 8.1 Hz, 1H), 7.10 (d, *J* = 8.1 Hz, 1H), 6.84 (s, 1H), 4.62 (dt, *J* = 12.4, 6.5 Hz, 1H), 3.90 (d, *J* = 10.0 Hz, 1H), 3.78 (dt, *J* = 11.0, 6.2 Hz, 1H), 3.34 (s, 2H), 3.14–2.96 (m, 2H), 2.29 (dd, *J* = 13.4, 7.0 Hz, 1H);^13^C NMR (101 MHz, DMSO-*d*_6_) δ 179.55, 144.93, 139.29, 135.14, 134.96, 130.44, 128.52, 127.64, 125.12, 122.17, 110.52, 69.35, 68.96, 62.52, 53.28, 34.56, 32.64; [Anal. Calcd. for C_19_H_17_ClN_2_O_3_S_2_: C, 54.22; H, 4.07; Cl, 8.42; N, 6.66; Found: C, 54.33; H, 4.10; N, 6.76]; LC/MS (ESI, *m/z*): found 421.12 [M+H]^+^; Exact Mass: 420.04.

### 3.2. Biological Activity Assays

The protocol for the biological activity assay is provided in the Supplementary Materials.

### 3.3. Molecular Docking

The protocol for the molecular docking study is provided in the Supplementary Materials.

### 3.4. ADMET Analysis

The protocol for the ADMET analysis is provided in the Supplementary Materials.

### 3.5. Molecular Dynamic Simulation

The protocol for the molecular dynamics simulation is provided in the Supplementary Materials.

## 4. Conclusions

Here, a detailed design and synthesis of spirooxindole-based phenylsulfonyl moiety compounds are provided and their *in vitro* antiviral activity was evaluated against pandemic SARS-CoV-2 and MERS-CoV with multiple sporadic human infections. Based on the preliminary screening of the anti-coronaviral activity of the tested compounds, compounds **4k, 4c**, and **4i** showed high safety and high-to-moderate anti-coronavirus activities. Synergistic combinations of the three compounds against SARS-CoV-2 displayed a more potent inhibitory activity. These promising combinations with high selectivity indices are recommended for further *in vitro* and *in vivo* preclinical studies. The determination of dosage form will be decided through pharmacodynamics and pharmacokinetic studies in the preclinical phases. However, based on the current physicochemical parameters virtually, these bioactive candidates could be administered in an oral dosage form, for example as capsules or tablets.

## Figures and Tables

**Table 1:** Chemical structures, cytotoxicity, antiviral activities of the tested compounds against SARS-CoV-2 and MERS-CoV as determined by MTT and plaque reduction assay, respectively.

Cpd	Chemical Structure	Cytotoxicity (CC_50_, µM)	Antiviral Activities			
			SARS-CoV-2		MERS-CoV	
			IC_50_ (µM)	SI	IC_50_ (µM)	SI
**4a**	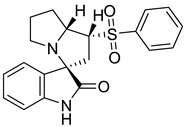	326	29	11.24	62	5.25
**4b**	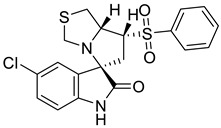	1045	194	5.38	105	9.95
**4c**	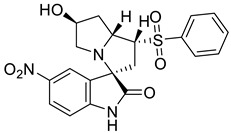	1095	18	60.83	65	16.84
**4d**	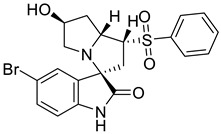	206	24	8.58	23	8.95
**4e**	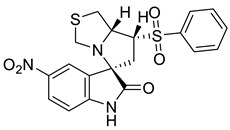	289	17	17	74	3.90
**4f**	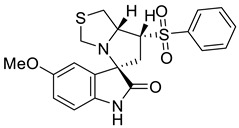	345	2251	<1	88	3.92
**4g**	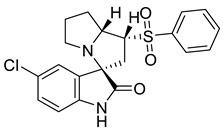	405	405	1	101	3.97
**4h**	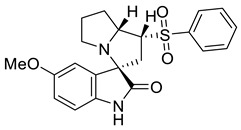	571	37	15.43	15	38.06
**4i**	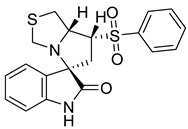	1989	34	58.5	11	180.81
**4j**	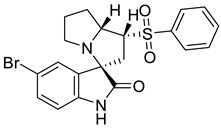	200	197	1.01	376	<1
**4k**	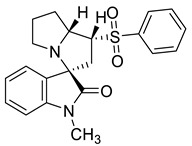	883	27	32.70	66	13.37
**4l**	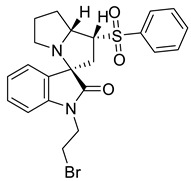	206	107	1.92	146	1.41
**4m**	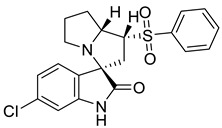	225	305	<1	191	1.17
**4n**	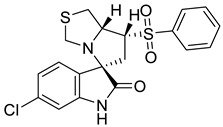	222	667	<1	15	14.8
**Remdesivir**		473.10	6.72	70.40	2.74	172.66

Abbreviations: “Cpd”, Compound; “CC_50_”, half maximal cytotoxic concentration; “IC_50_”, half maximal inhibitory concentration; “SI”, Selectivity index; Remdesivir as a drug control.

**Table 2:** In silico predicted ADMET properties of spirooxindole analogs.

Compounds	Mol. Weight g/mol	nHA	nHD	TPSA	LogP	Lipinski Rule	Acute Toxicity Rule	HIA	PAINS
**4c**	429.100	9	2	129.8	1.80	Accepted	0 alerts	Yes	0 alerts
**4i**	386.080	5	1	66.4	2.38	Accepted	0 alerts	Yes	0 alerts
**4k**	382.140	5	0	57.6	2.35	Accepted	0 alerts	Yes	0 alerts

## Data Availability

Not applicable.

## Supplementary Materials

The following supporting information can be downloaded at: https://www.mdpi.com/article/10.3390/ijms231911861/s1, Protocols for the in vitro biological activity assays, molecular docking, ADMET Analysis, and Molecular Dynamic Simulation. Figures S1–S24: NMR spectra (^1^H and ^13^C); Figures S25–S27: Cytotoxicity, Antiviral activities against SARS-CoV-2 and MERS-CoV. Figures S28 and S29: Molecular docking optimization.
