# Supplementary material for: Design, Synthesis and In Vitro Evaluation of Spirooxindole-Based Phenylsulfonyl Moiety as a Candidate Anti-SAR-CoV-2 and MERS-CoV-2 with the Implementation of Combination Studies
Source: Int J Mol Sci. 2022 Oct 6;23(19):11861. doi: 10.3390/ijms231911861 (PMC9569468; doi:10.3390/ijms231911861)
Supplement: Supplementary file 1 [file ijms-23-11861-s001.zip › ijms-1924985-supplementary.pdf]

SUPPORTING INFORMATION

Design, Synthesis and in Vitro Evaluation of Spirooxindole-Based Phenylsulphonyl Moiety as a Candidate Anti-SAR-CoV-2 and MERS-CoV-2 with the Implementation of Combination Studies

Assem Barakat [1,*], Ahmed Mostafa [2], M. Ali[1], Abdullah Mohammed Al-Majid [1], Luis R. Domingo[3], Omnia Kutkat [2], Yassmin Moatasim [2], Komal Zia [4], Zaheer Ul-Haq [4, 5] and Yaseen A. M. M. Elshaier[6,*]

1 Department of Chemistry, College of Science, King Saud University, P. O. Box 2455, Riyadh 11451, Saudi Arabia
2 Center of Scientific Excellence for Influenza Viruses, National Research Centre, Giza 12622, Egypt
3 Department of Organic Chemistry, University of Valencia, Dr. Moliner 50, 46100 Burjassot, Valencia, Spain
4 Dr. Panjwani Center for Molecular Medicine and Drug Research, International Center for Chemical and Biological Sciences, University of Karachi, Karachi-75270, Pakistan
5 H.E.J. Research Institute of Chemistry, International Center for Chemical and Biological Sciences, University of Karachi, Karachi-75270, Pakistan
6 Department of Organic and Medicinal Chemistry, Faculty of Pharmacy, University of Sadat City, Menoufiya 32958, Egypt
* Correspondence: ambarakat@ksu.edu.sa; Tel.: +966-11467-5901; Fax: +966-11467-5992 (A.B.). Yaseen.elshaier@fop.usc.edu.eg (Y.A.M.M.E.).

1.1. Biology

1.1.1. MTT cytotoxicity assay (CC_{50})

Samples were diluted with Dulbecco's Modified Eagle's Medium (DMEM). Stock solutions of the test compounds were prepared in 10 % DMSO in dd H_2O. The cytotoxic activity of the extracts was tested in Vero E6 cells by using the 3-(4,5-dimethylthiazol -2-yl)-2, 5-diphenyltetrazolium bromide (MTT) method. Briefly, the cells were seeded in 96 well-plates (100 µl/well at a density of 3×10^5 cells/ml) and incubated for 24 h at 37°C in 5%CO_2. After 24 h, cells were treated with various concentrations of the tested compounds in triplicates. After further 24 h, the supernatant was discarded and cell monolayers were washed with sterile phosphate buffer saline (PBS) 3 times and MTT solution (20 µl of 5 mg/ml stock solution) was added to each well and incubated at 37°C for 4 h followed by medium aspiration. In

each well, the formed formazan crystals were dissolved with 200 µl of acidified isopropanol (0.04 M HCl in absolute isopropanol = 0.073 ml HCL in 50 ml isopropanol). Absorbance of formazan solutions were measured at λmax 540 nm with 620 nm as a reference wavelength using a multi-well plate reader. The half maximal viral inhibitory (IC_{50}) concentrations of the compounds were measured as previously described. The cytotoxicity of various concentrations compared to the untreated cells was determined using nonlinear regression analysis by plotting log inhibitor concentrations versus normalized responses.

1.1.2. Plaque reduction assay

To assess the preliminary antiviral activity of the studied spiro-compounds, the plaque reduction assay was performed in a six well plate where Vero E6 cells (10^5 cells ⁄ml) were cultivated for 24 h at 37°C. Briefly, the Middle East respiratory syndrome-related coronavirus isolate NRCE-HKU270 or hCoV-19/Egypt/NRC-3/2020 "NRC-03-nhCoV" virus was diluted to give 10^2 PFU⁄well and mixed with the safe concentration of the tested compounds and incubated for 1 hour at 37°C before being added to the cells. Growth medium was removed from the cell culture plates and the cells were inoculated with (100 µl/well) virus with the tested compounds, After 1 h contact time for virus adsorption, 3 ml of DMEM supplemented with 2% agarose and the tested compounds was added onto the cell monolayer, plates were left to solidify and incubated at 37°C till formation of viral plaques (3 to 4 days). Formalin (10%) was added for two hours then plates were stained with 0.1 %crystal violet in distilled water. Control wells were included where untreated virus was incubated with Vero E6 cells and finally plaques were counted and percentage reduction in plaques formation in comparison to control wells was recorded as following

% inhibition= viral count (untreated) - viral count (treated)/viral count (untreated) x 100

1.1.3. Inhibitory concentration (IC_{50})

To investigate the anti-SARS-CoV-2 activity with safe concentrations of the selected compound, MTT cytotoxicity assay was used to determine the half maximal cytotoxic concentration (CC_{50}) for each compound in Vero-E6 as described previously[20]. The half maximal cytotoxic concentrations "CC_{50}" were calculated using nonlinear regression analysis of GraphPad Prism software (version 5.01) by plotting log inhibitor versus normalized response (variable slope). Afterwards, different safe concentrations of the selected compounds were applied in triplicate together with NRC-03-nhCoV virus (100 $TCID_{50}$) in confluent Vero-E6 monolayers against virus

control (virus only) and cell control as previously described[23]. Approximately 72 h post-treatment, the cell monolayers were fixed with 100 µl of 4% paraformaldehyde for 1 h and stained with 0.1% crystal violet in distilled water for 15 min at RT. The crystal violet dye was then dissolved using 100 µl absolute methanol per well and the optical density of the color is measured at 570 nm using Anthos Zenyth 200rt plate reader (Anthos Labtec Instruments, Heerhugowaard, Netherlands). The half maximal inhibitory concentrations (IC_{50}) of the compound is that required to reduce the virus-induced cytopathic effect (CPE) by 50%, relative to the cell and virus controls. The IC_{50} were estimated using nonlinear regression analysis of GraphPad Prism software (version 5.01) by plotting log inhibitor versus normalized response (variable slope).

1.2. Molecular Docking

The X-ray crystal structure coordinate of SARS-CoV-2 RNA-dependent RNA polymerase (RdRp) and MERS-CoV viral proteins nsp5 (PDB:ID: 4ylu) were retrieved from PDB (PDB ID: 6m71) with its co-crystallized bound ligand. The docking study was performed using openEye scientific software version 2.2.5 (SantaFe, NM (USA), http://www.eyesopen.com), academic license (The Laboratory of Yaseen A. M. Mohamed Elshaier. A virtual library of the synthesized compounds was used, and their energies were minimized using the MMFF94 force field, followed by the generation of multi-conformers using the OMEGA application. The library was compiled in one file by Omega. The target proteins were retrieved from PDB and the created receptor was operated by OeDocking application. Both the ligand input file and the receptor input file were subjected to FRED to implement the molecular docking study. Multiple scoring functions were engaged to predict energy profile of the ligand-receptor complex. The vida application was used as a visualization method The dimension for created box of receptors were as follow: For SARS-CoV-2 protein (PDB ID:6m71): Box volume: 8484 A, dimension 15.33 A° ×20.00 A° ×27.67 A°. For MERS-CoV-2: (PDB ID:4ylu): Box volume: 5544 A, dimension 21.00 A°×18.00 A° ×14.67 A°.

1.3. ADMET Analysis

The physiochemical and pharmacodynamic properties of the selected potential compounds was achieved by ADMETlab 2.0 webserver [55]. The SMILES notation of the compounds were subjected to submission page of ADMETlab to predict the ADMET properties.

1.4. Molecular Dynamics Simulation

In this study, we used a previously established protocol by Khan *et. al* [56] for MD simulation. Briefly, all the simulations were carried out by Amber20 using GPU acceleration through pmemd.cuda. The systems were solvated in a 8 Å TIP3P water box and neutralized with Na^{+} ions by tLeap module of Amber 20. The prepared systems were minimized by steepest descent and conjugate gradient algorithms. The systems were than equilibrated in NVT and NPT ensemble. The final production of 100ns was carried out and the Root Mean Square Deviation (RMSD) and Root Mean Square Fluctuation (RMSF) were calculated by CPPTRAJ to evaluate the dynamic stability of the complexes.

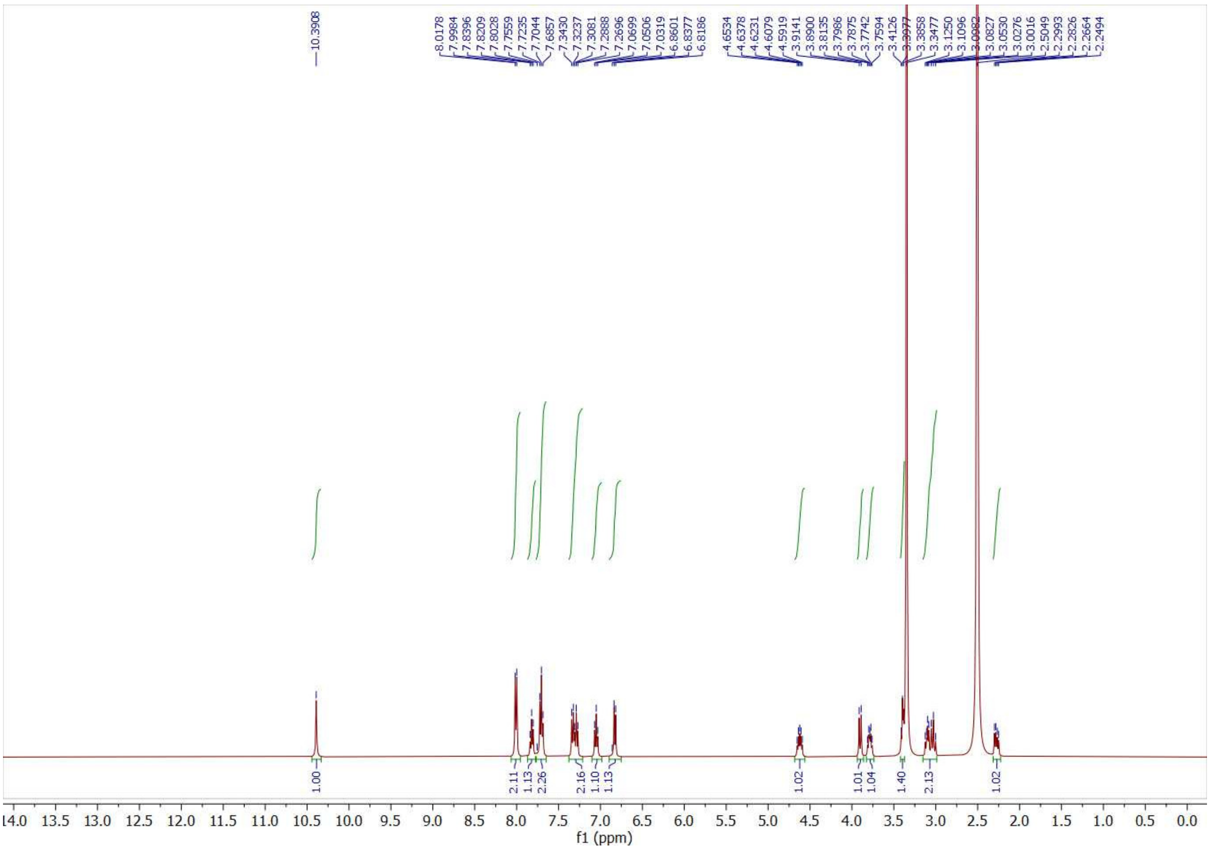

Figure S1: ^{1}HNMR of Compound **4a**

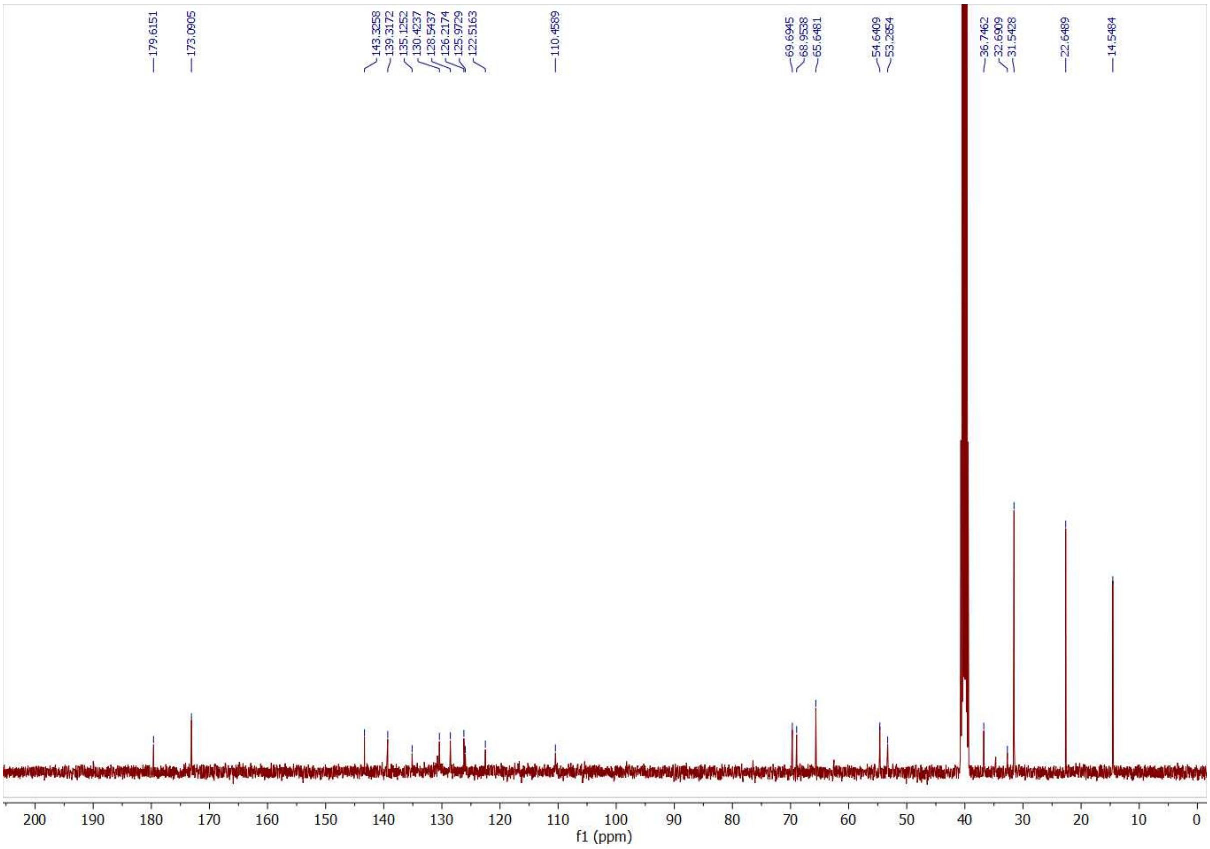

Figure S2: ^{13}CNMR of Compound **4a**

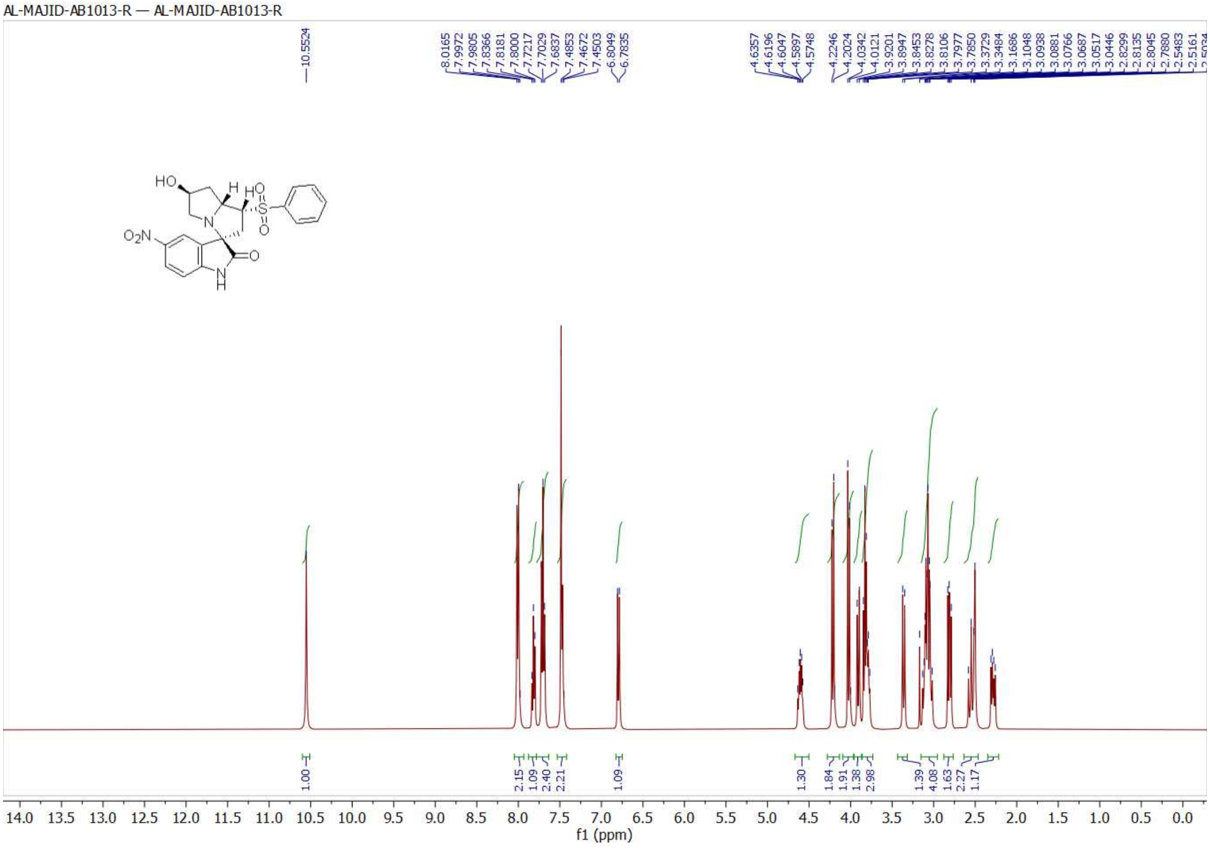

Figure S3: ^{1}HNMR of Compound **4c**

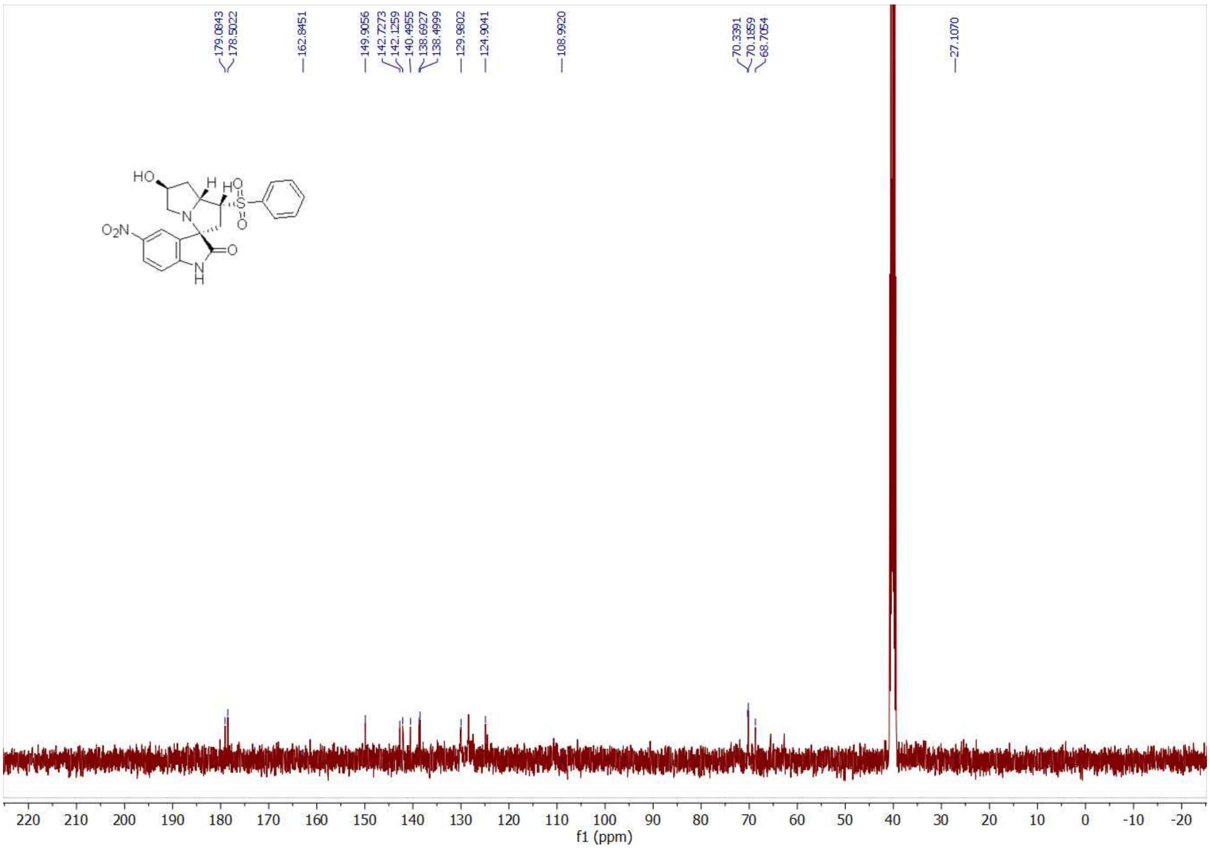

Figure S4: ^{13}CNMR of Compound **4c**

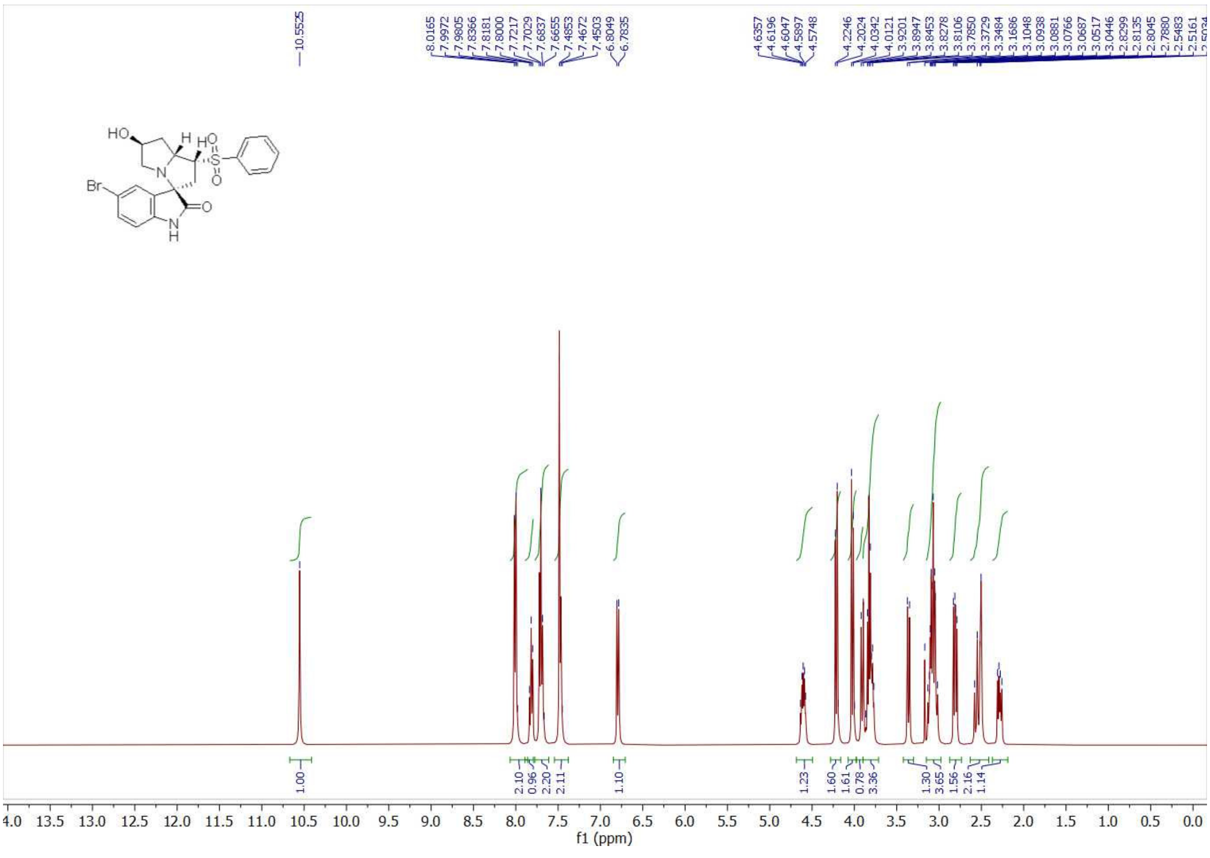

Figure S5: ^{1}HNMR of Compound **4d**

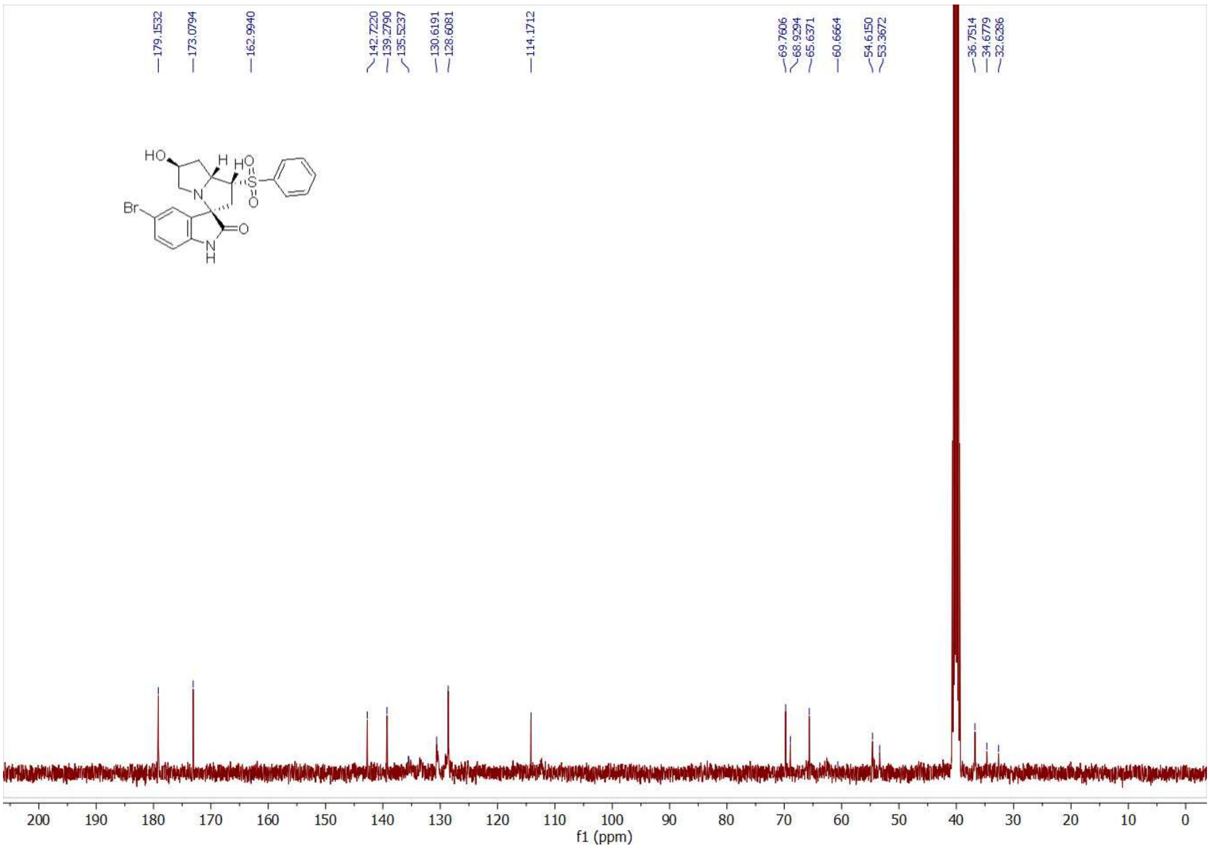

Figure S6: ^{13}CNMR of Compound **4d**

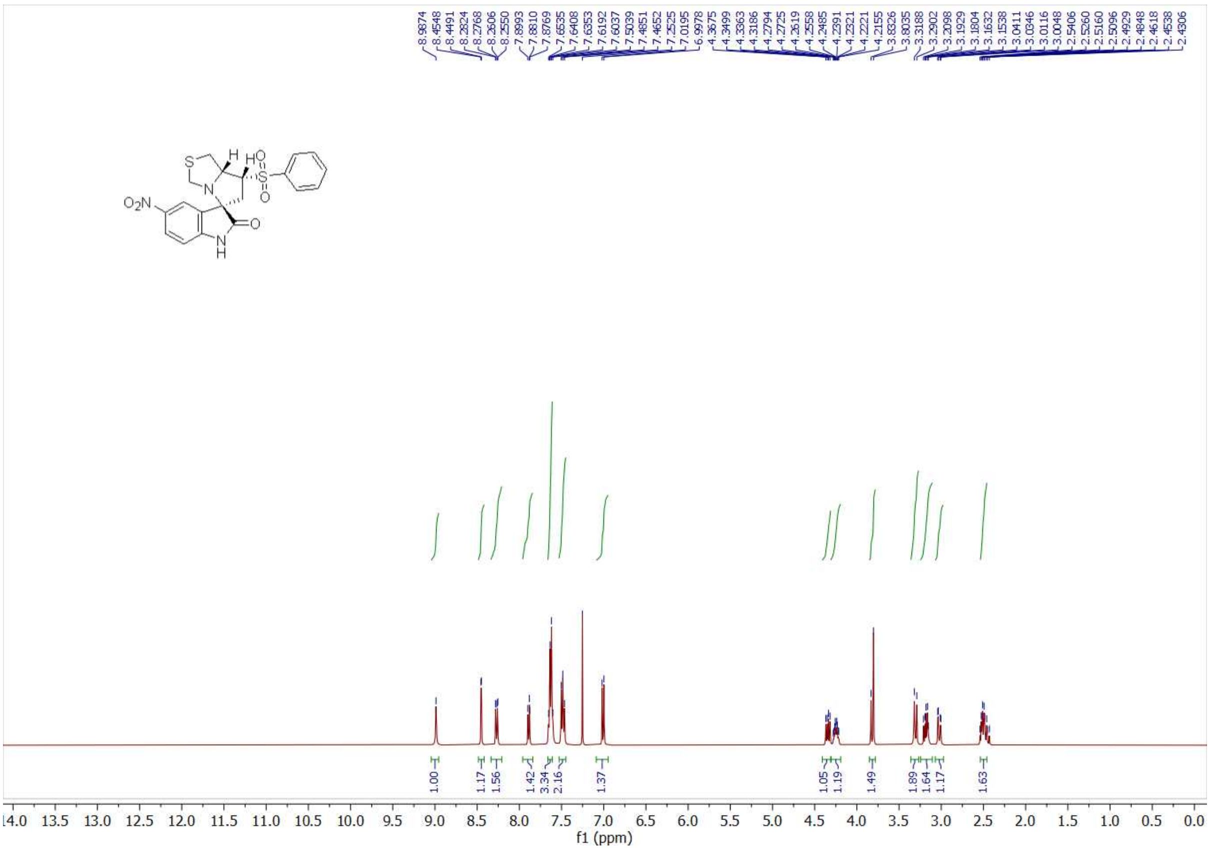

Figure S7: ^{1}HNMR of Compound **4e**

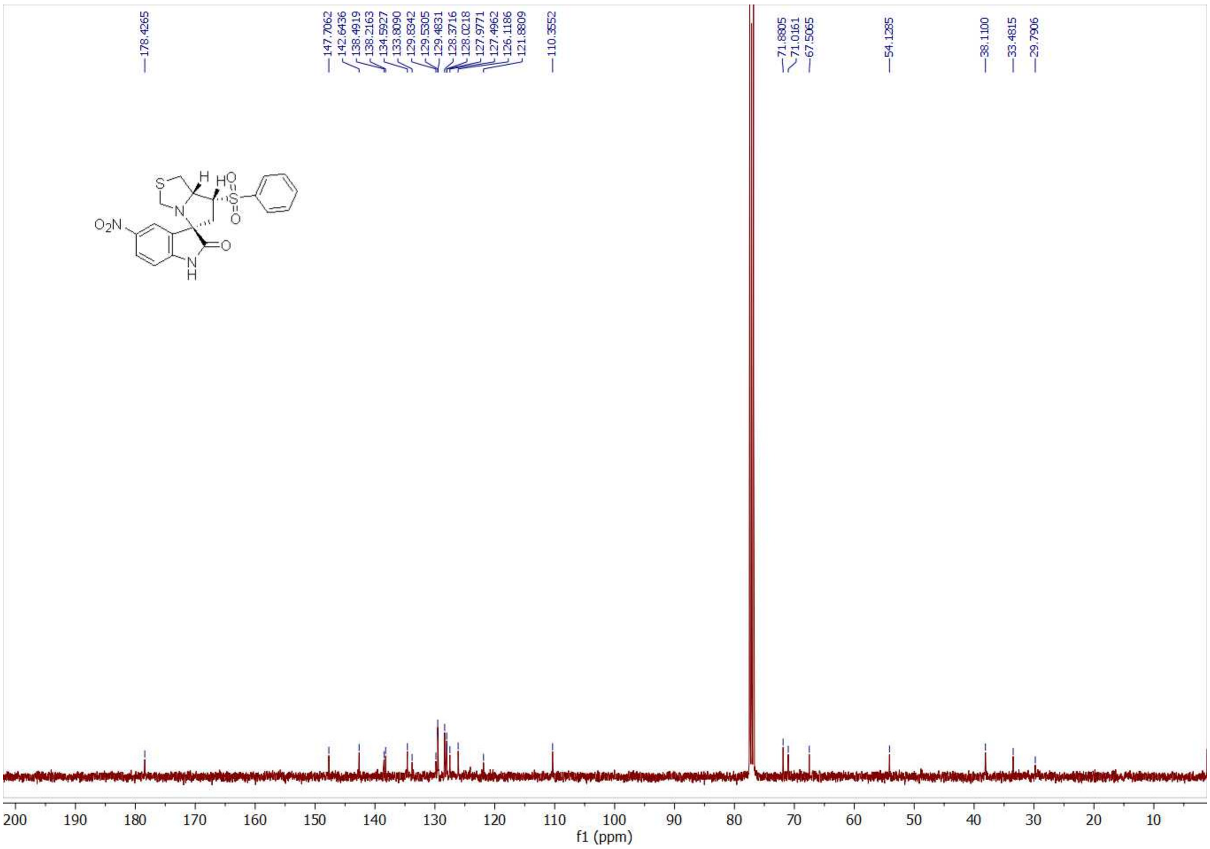

Figure S8: ^{13}CNMR of Compound **4e**

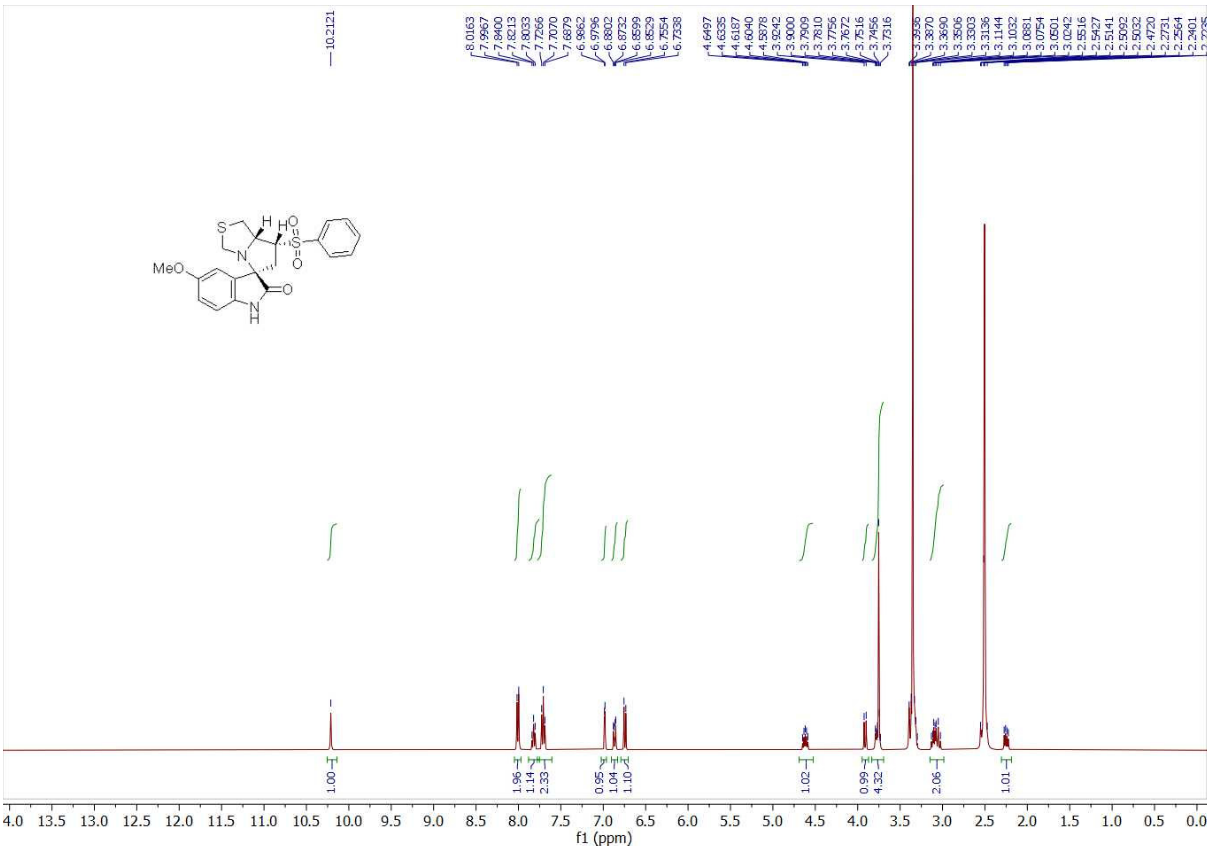

Figure S9: ^{1}HNMR of Compound **4f**

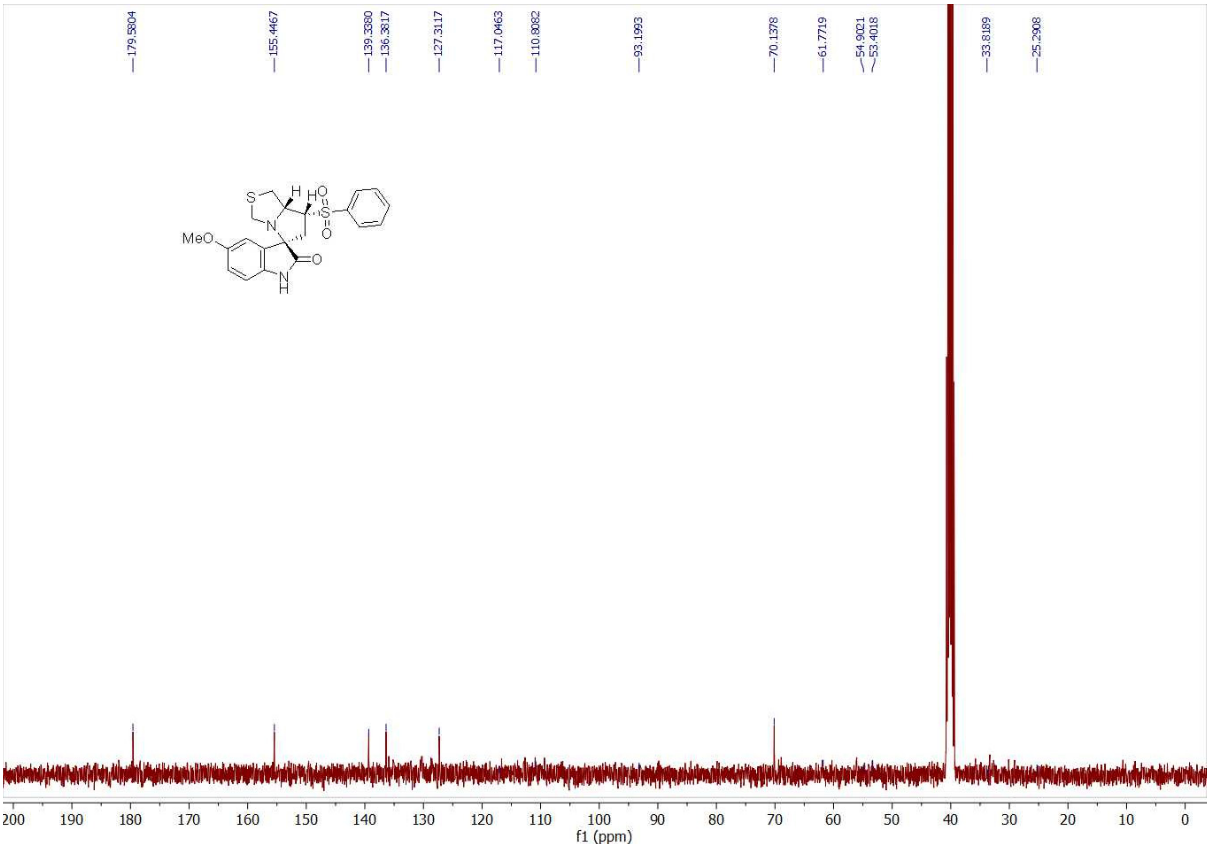

Figure S10: ^{13}CNMR of Compound **4f**

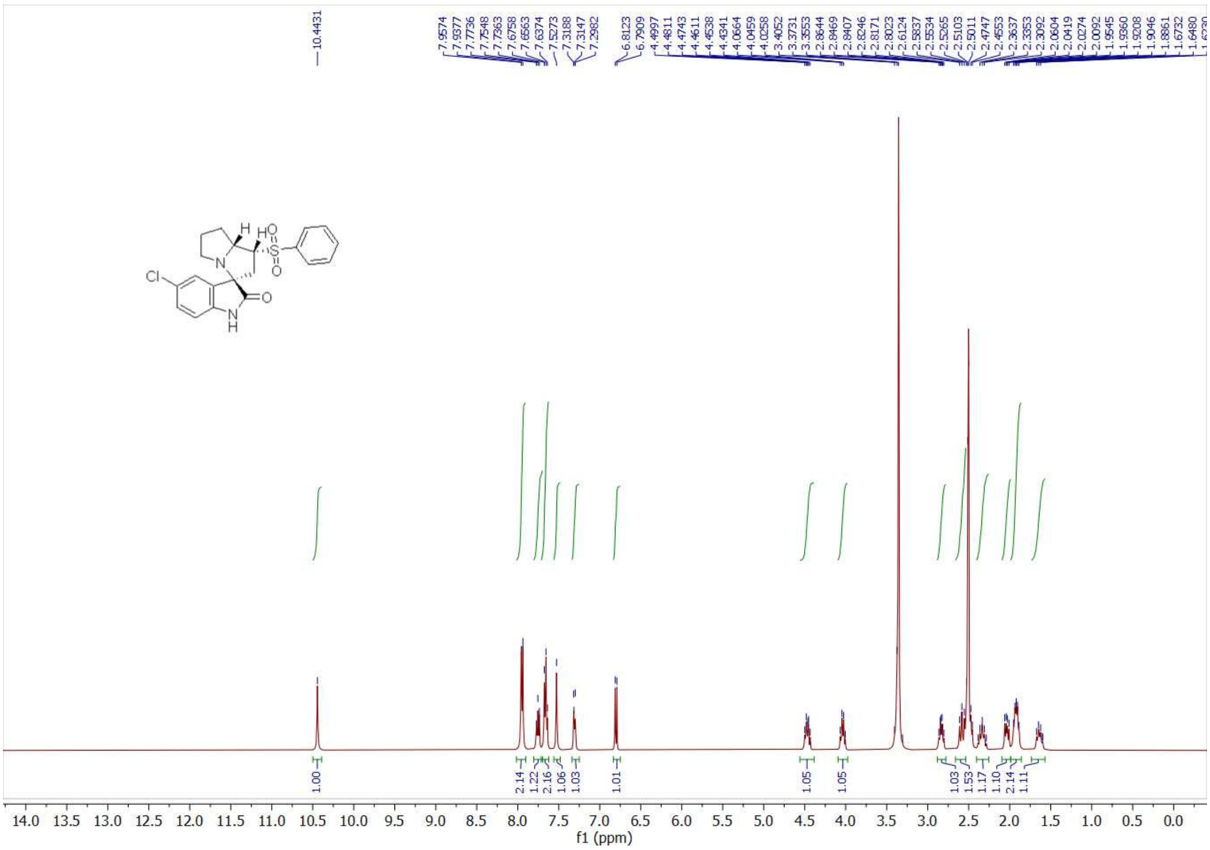

Figure S11: ^{1}HNMR of Compound **4g**

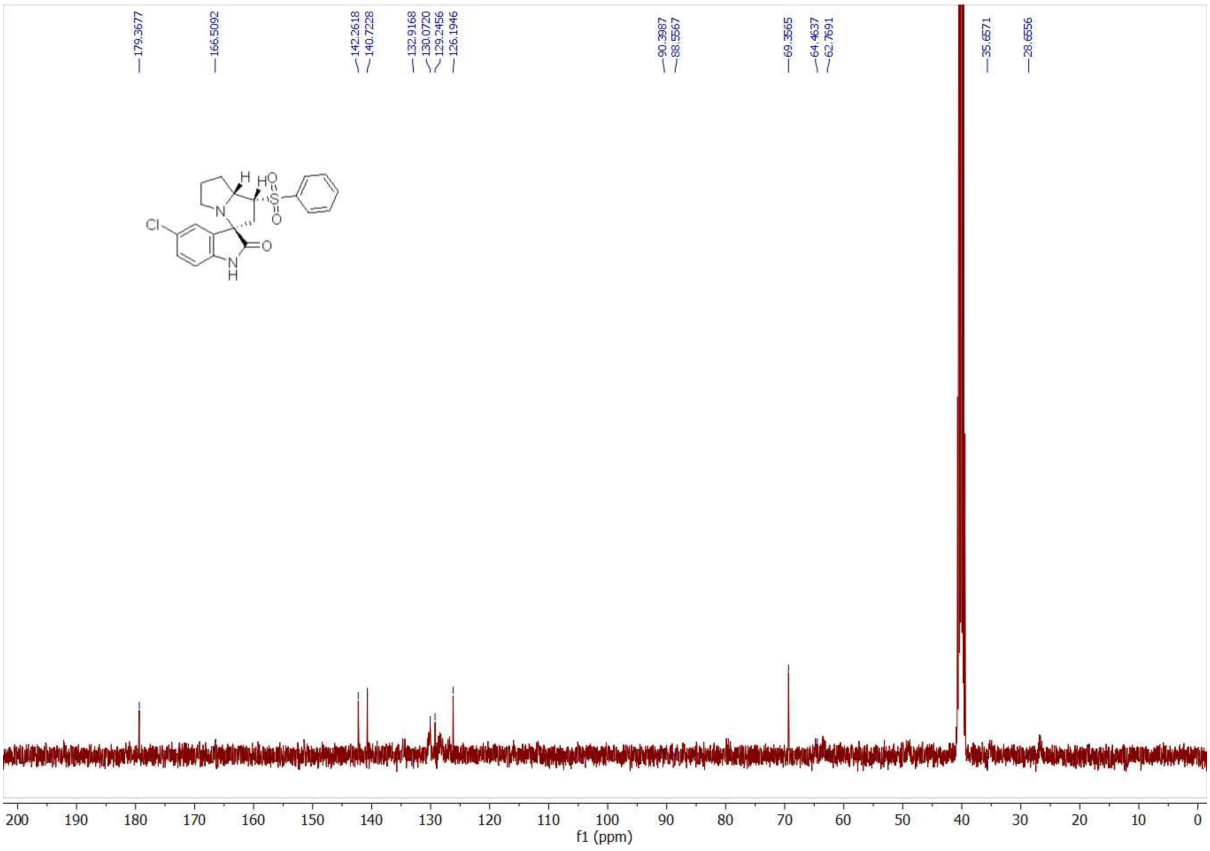

Figure S12: ^{13}CNMR of Compound **4g**

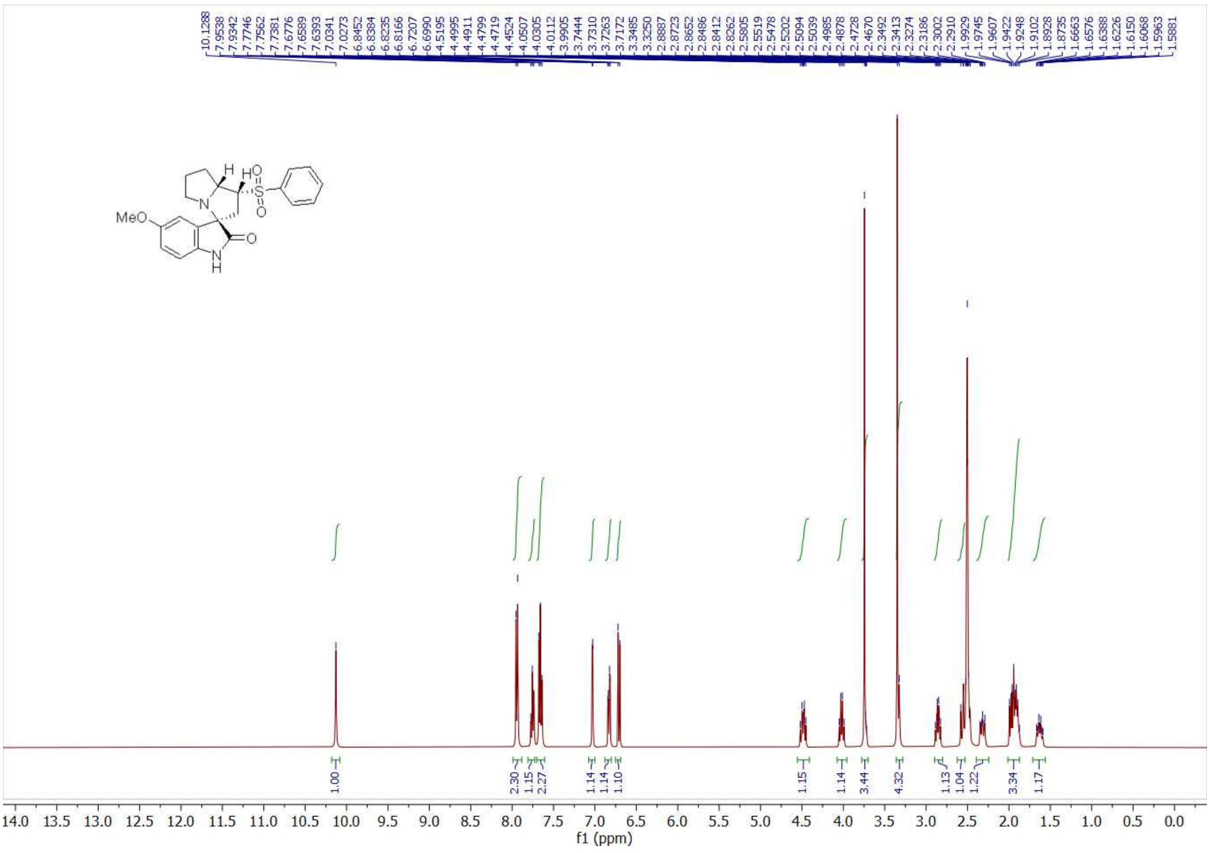

Figure S13: ^{1}HNMR of Compound **4h**

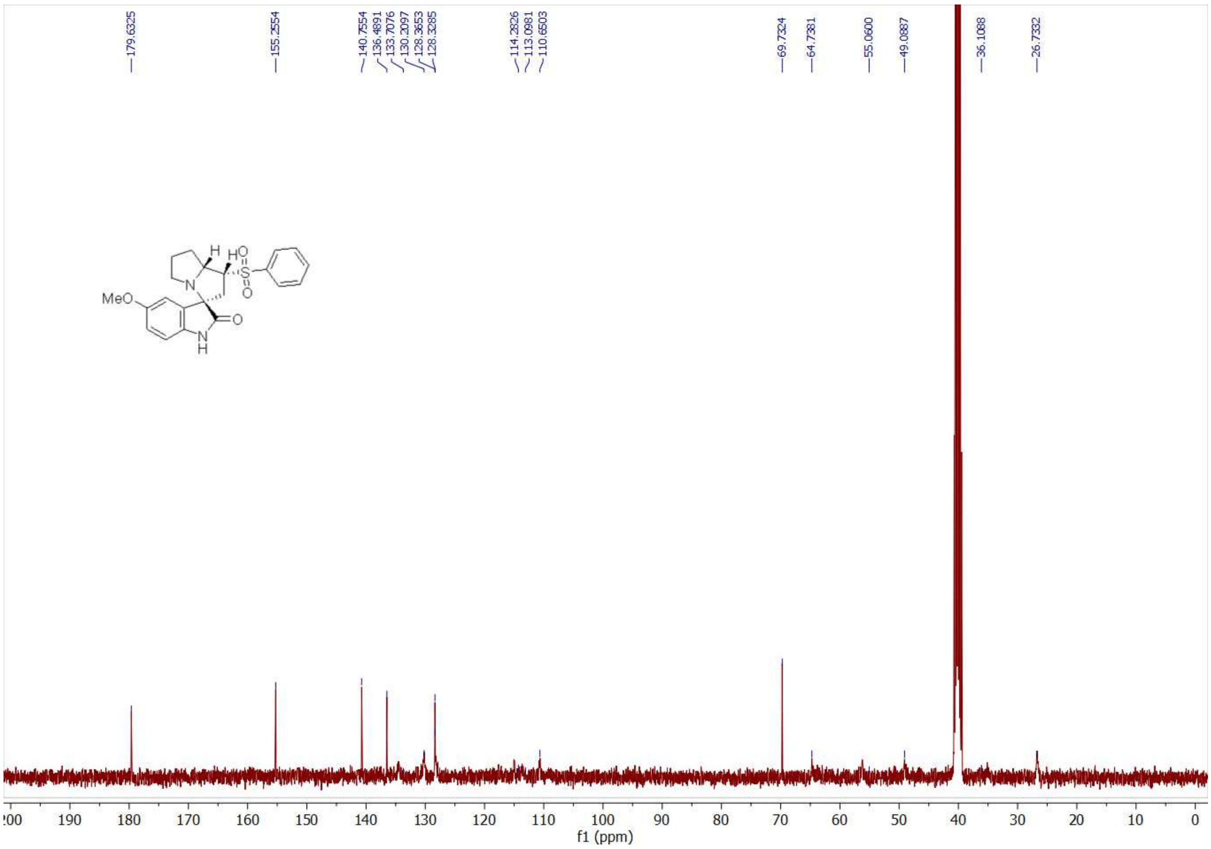

Figure S14: ^{13}CNMR of Compound **4h**

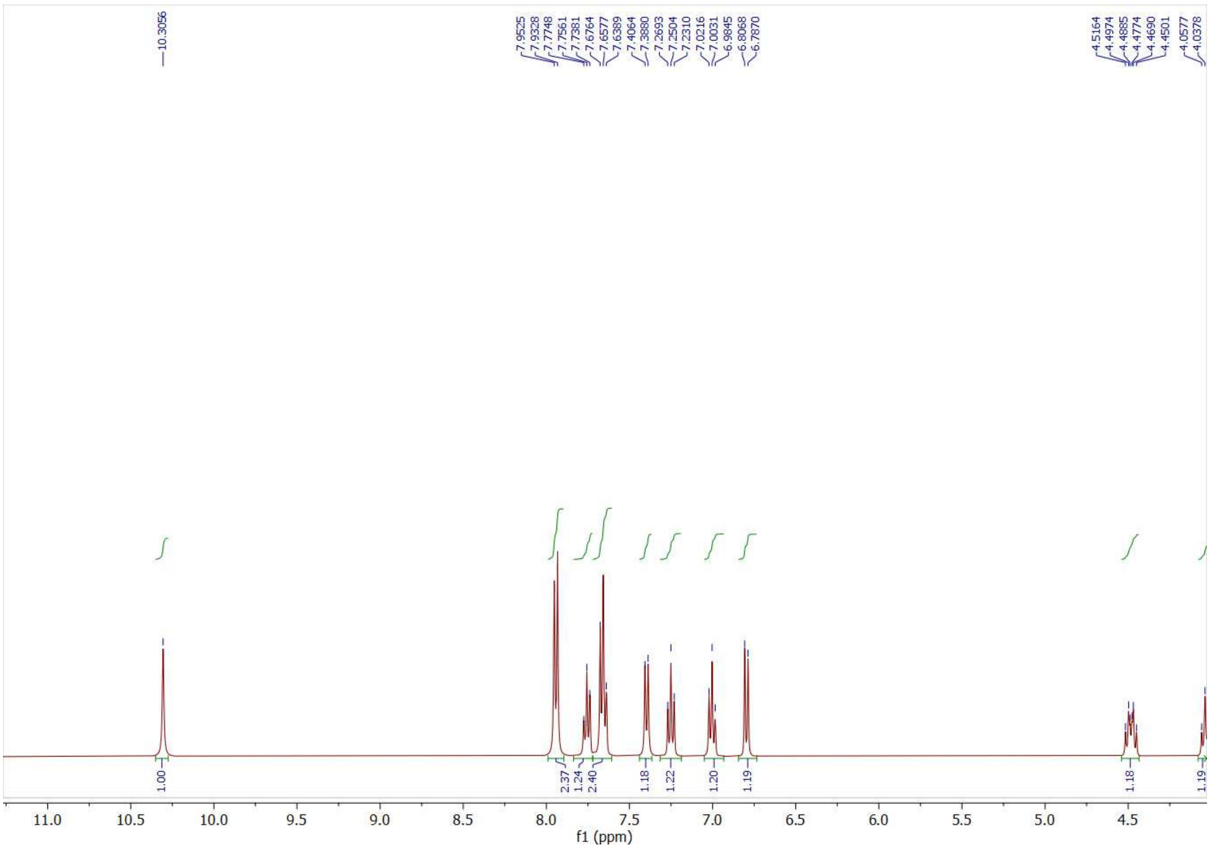

Figure S15: ^{1}HNMR of Compound **4i**

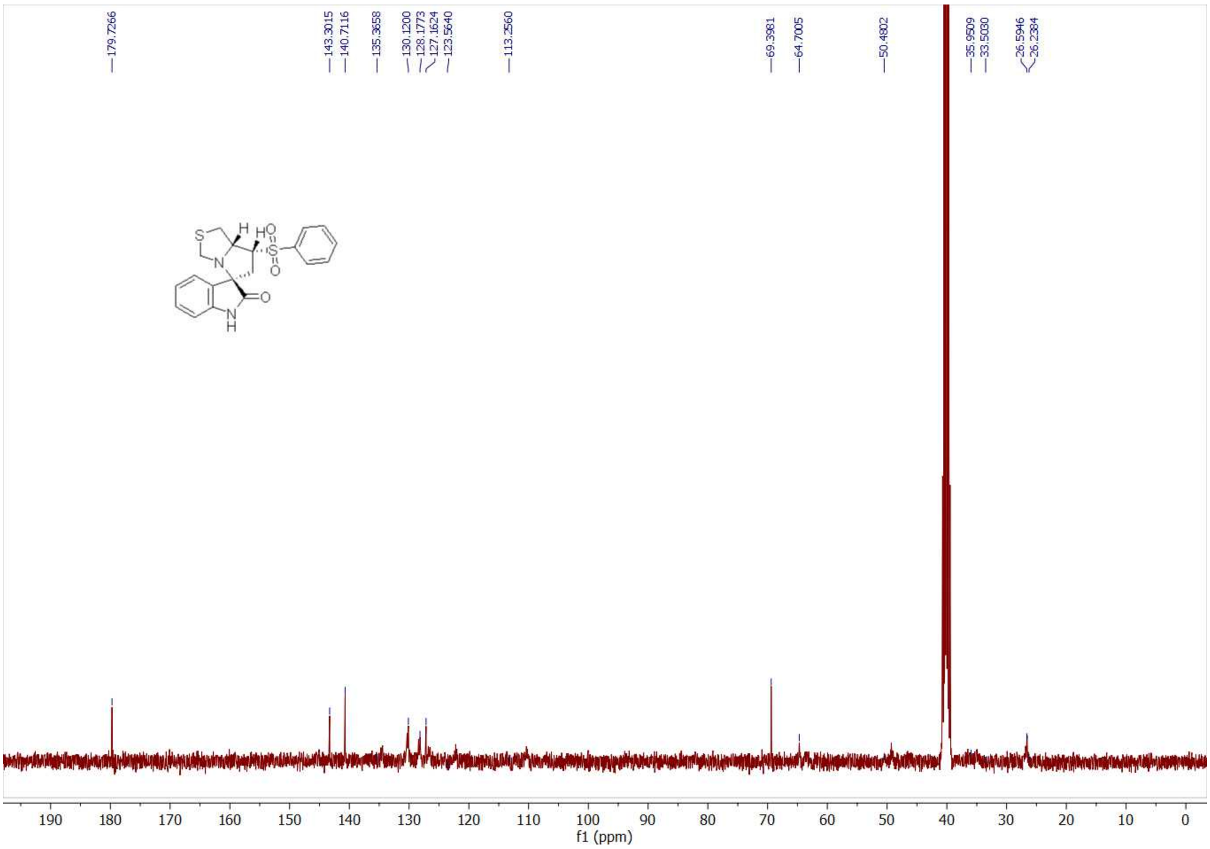

Figure S16: ^{13}CNMR of Compound **4i**

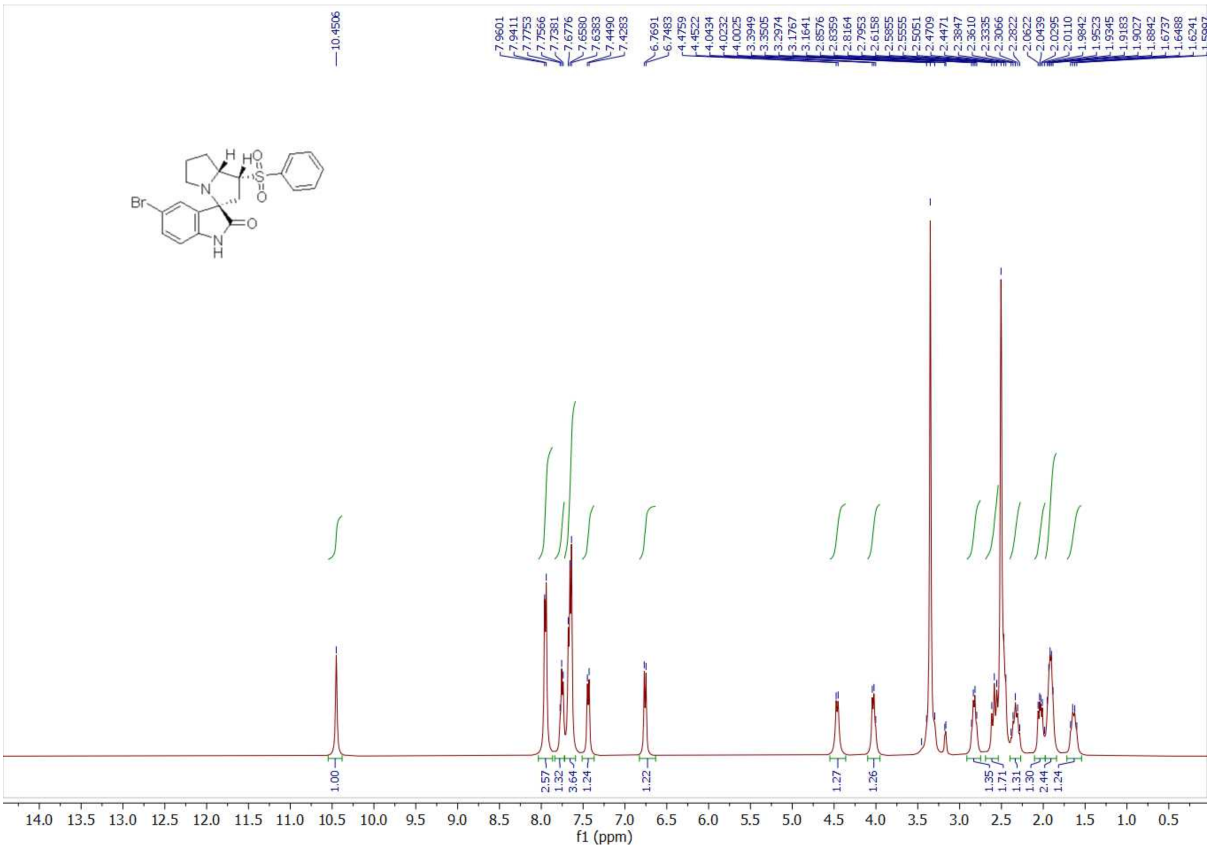

Figure S17: ^{1}HNMR of Compound **4j**

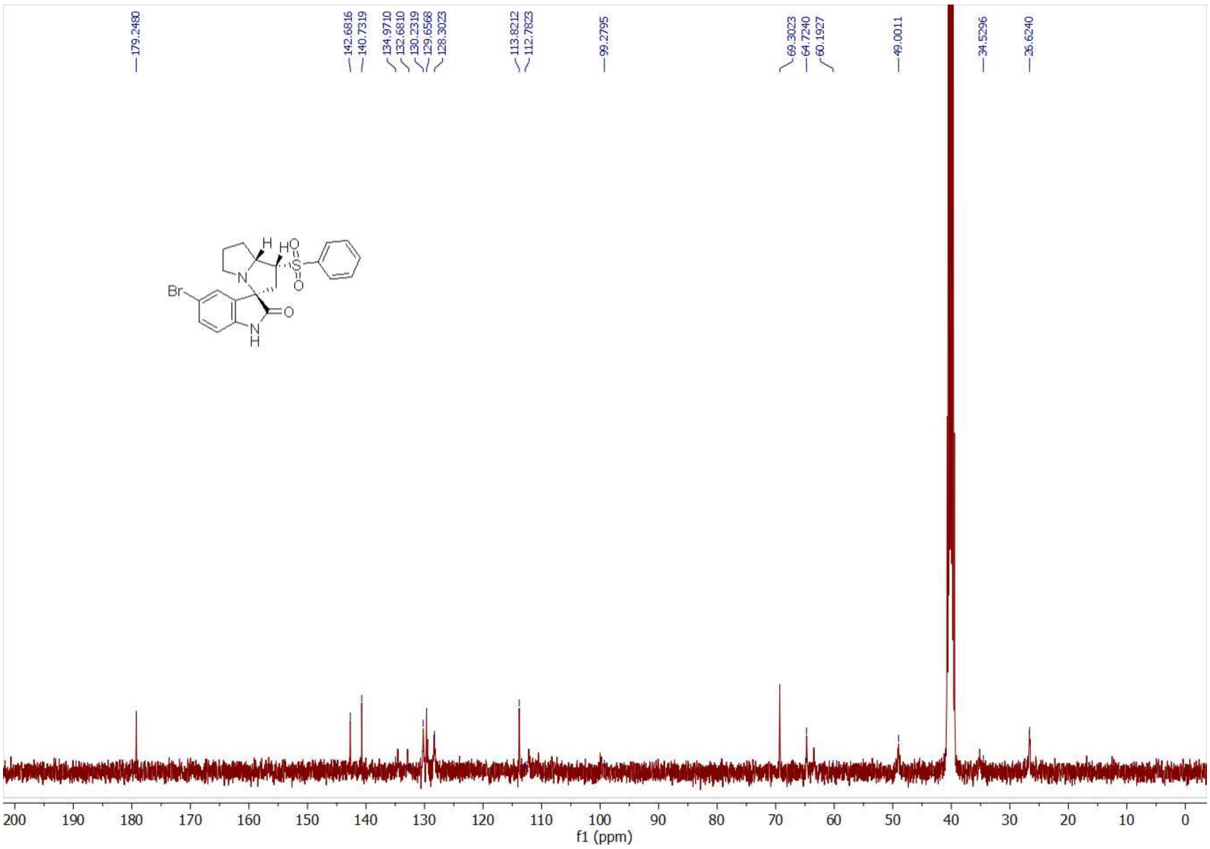

Figure S18: ^{13}CNMR of Compound **4j**

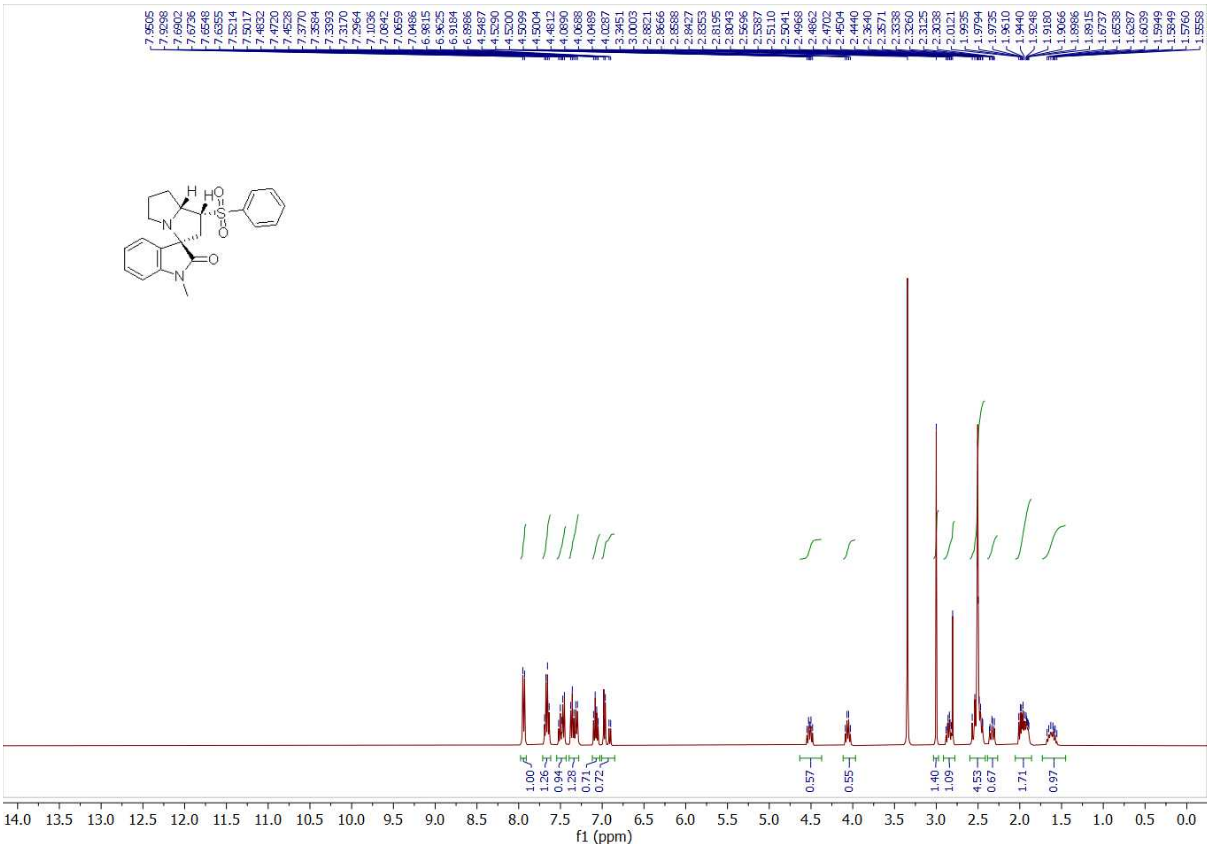

Figure S19: ^{1}HNMR of Compound **4k**

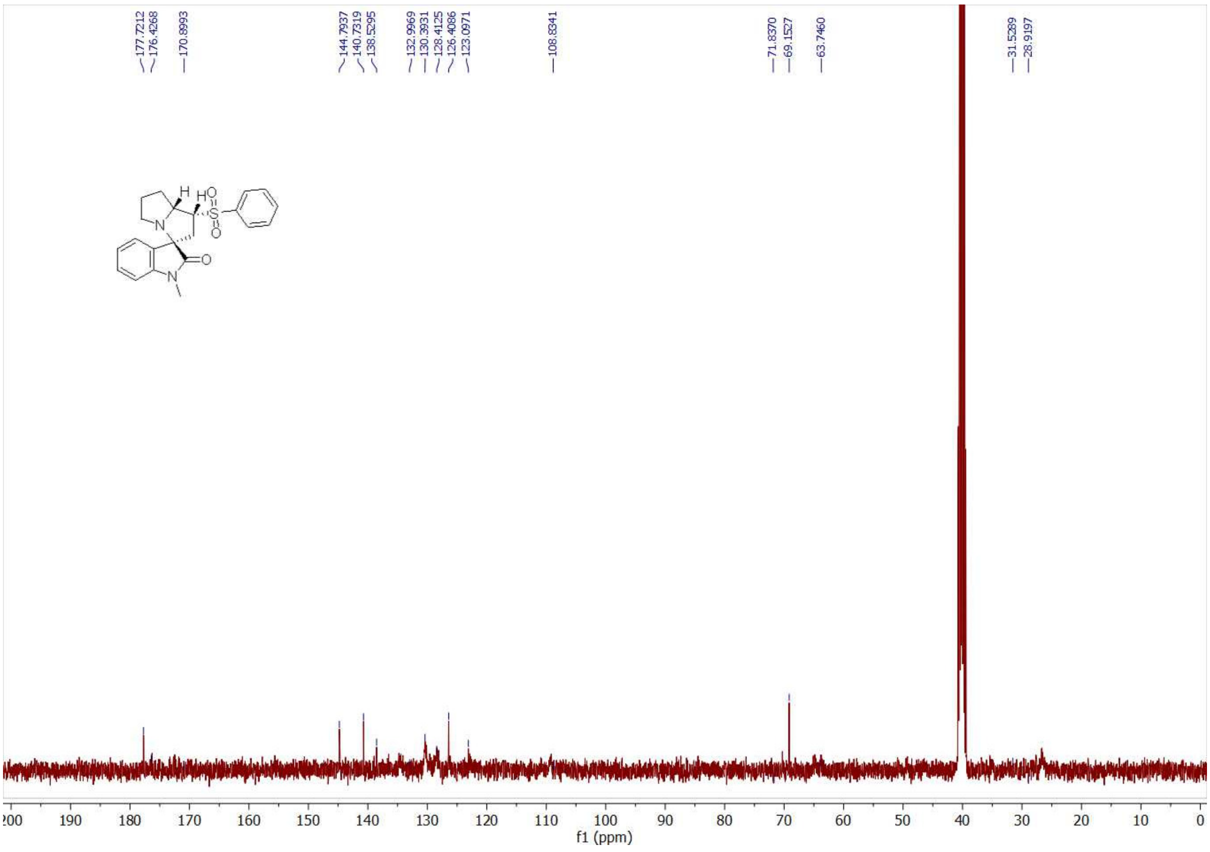

Figure S20: ^{13}CNMR of Compound **4k**

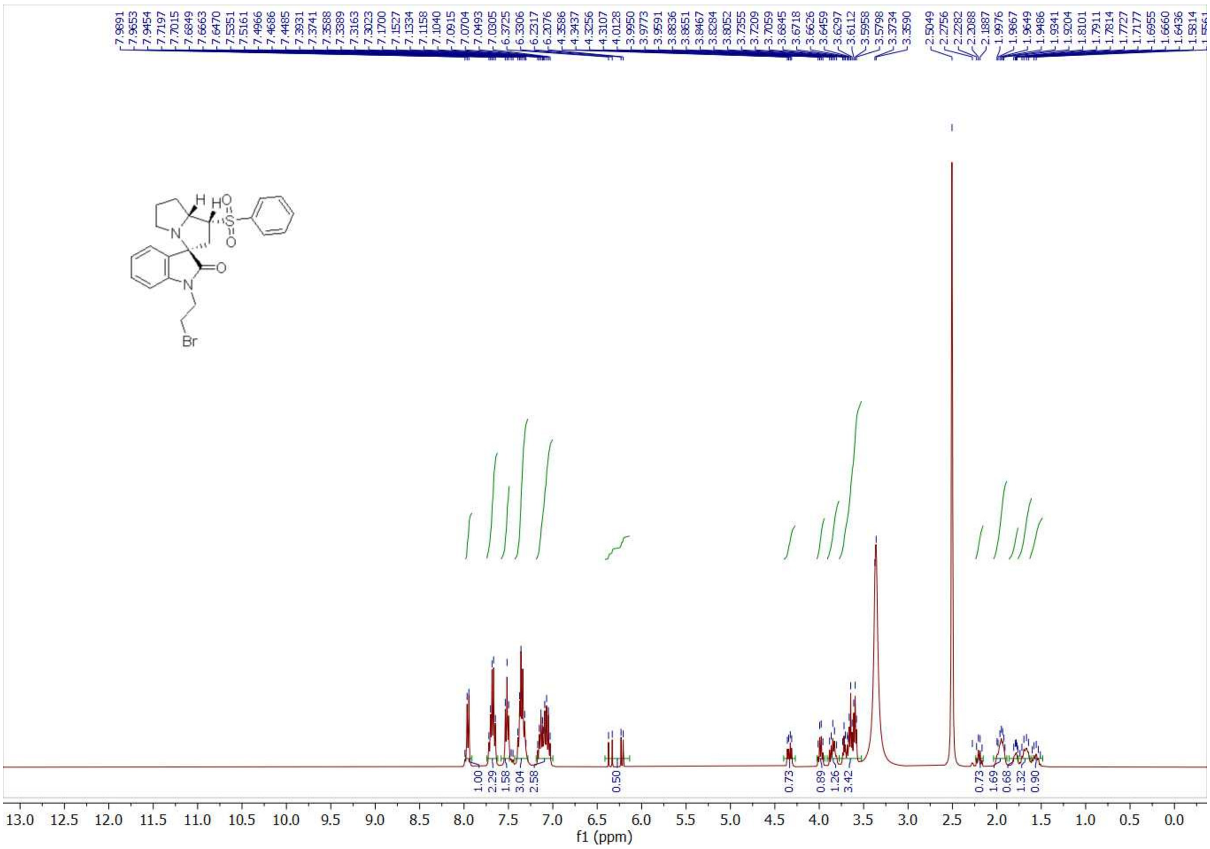

Figure S21: ^{1}HNMR of Compound **4l**

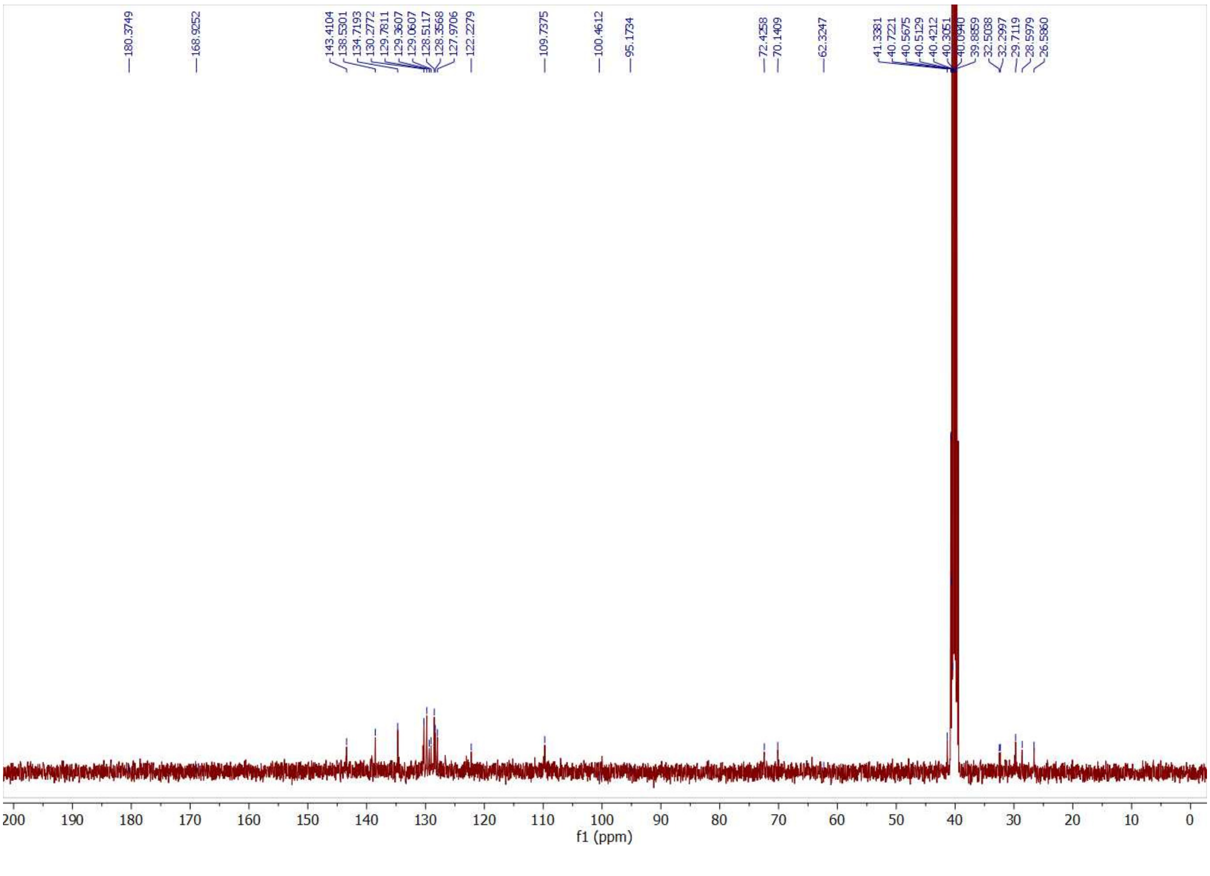

Figure S22: ^{13}CNMR of Compound **4l**

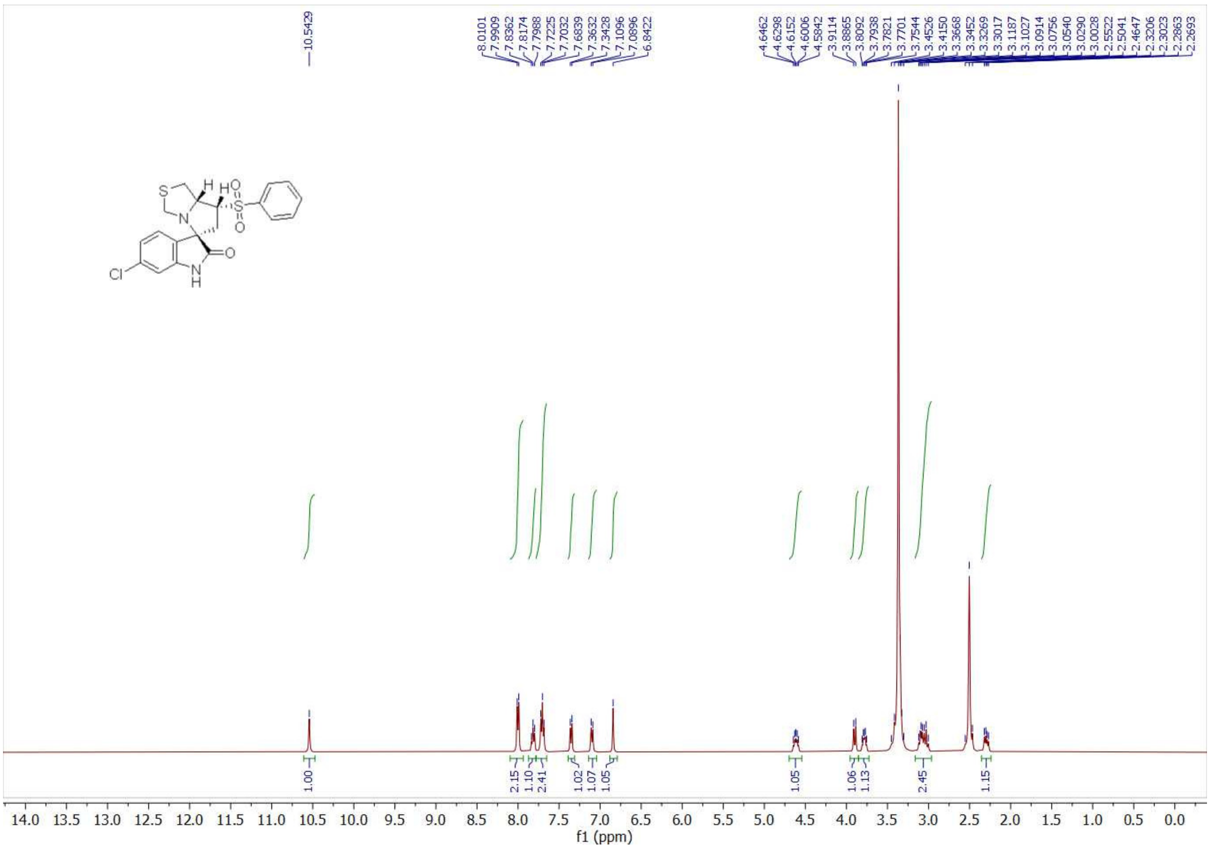

Figure S23: ^{1}HNMR of Compound **4n**

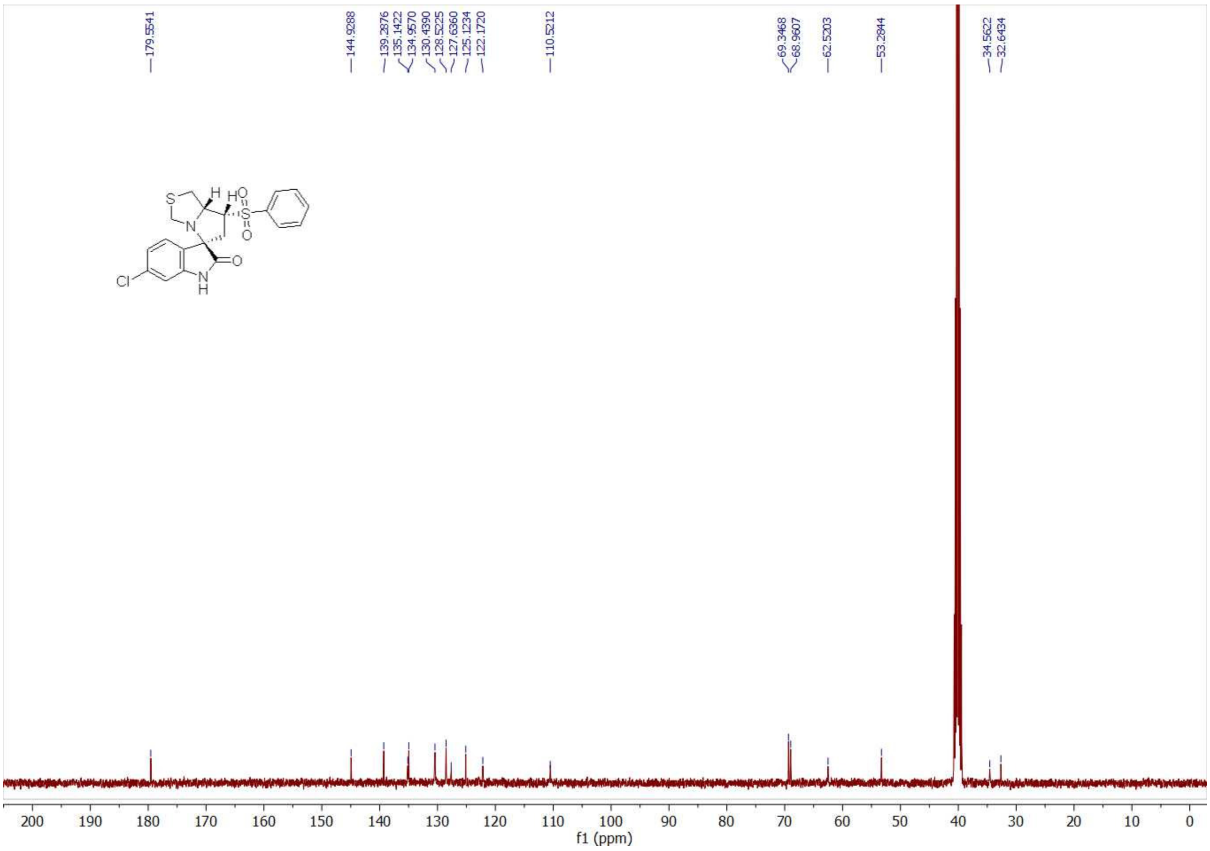

Figure S24: ^{13}CNMR of Compound **4n**

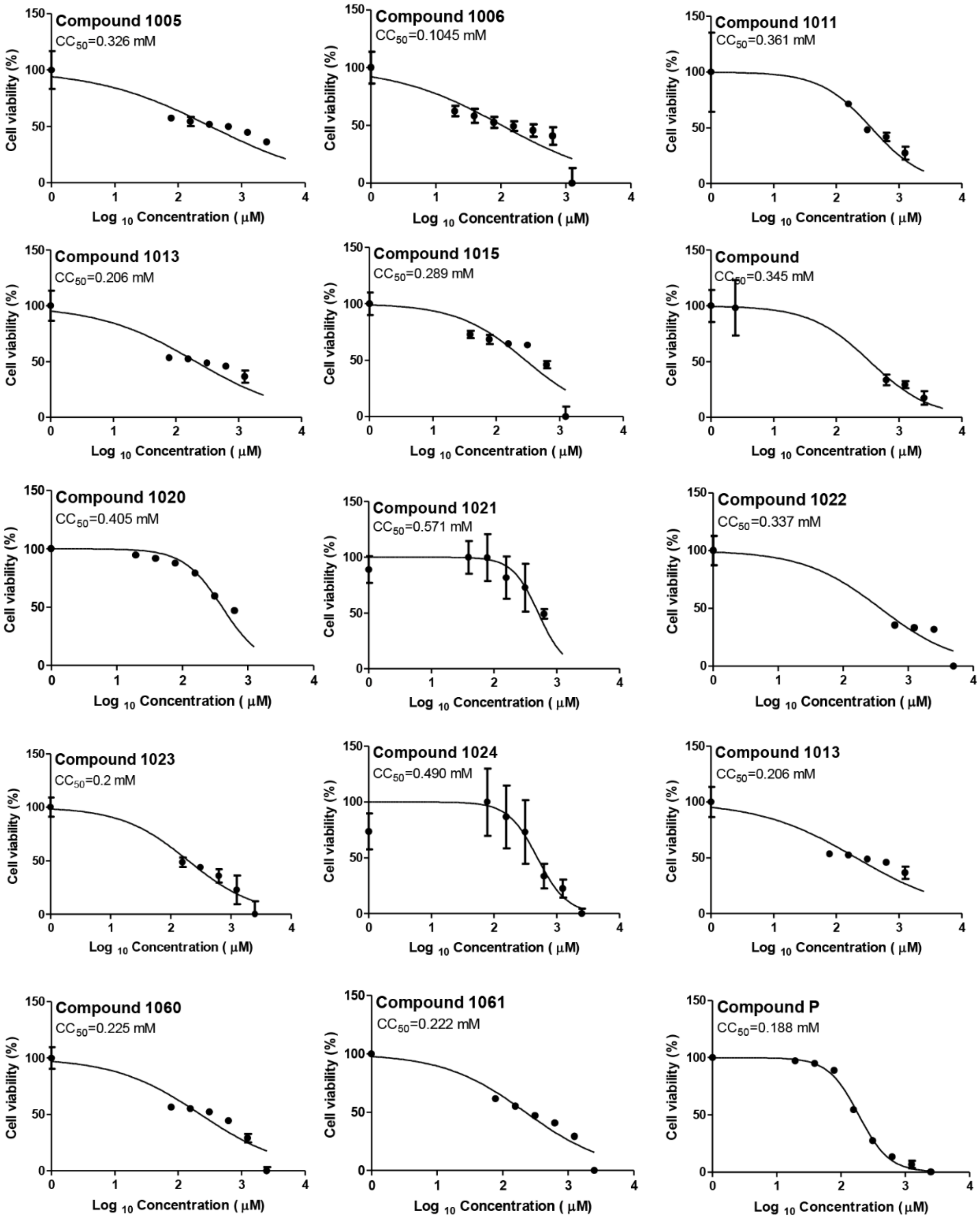

Figure S25: Cytotoxicity.

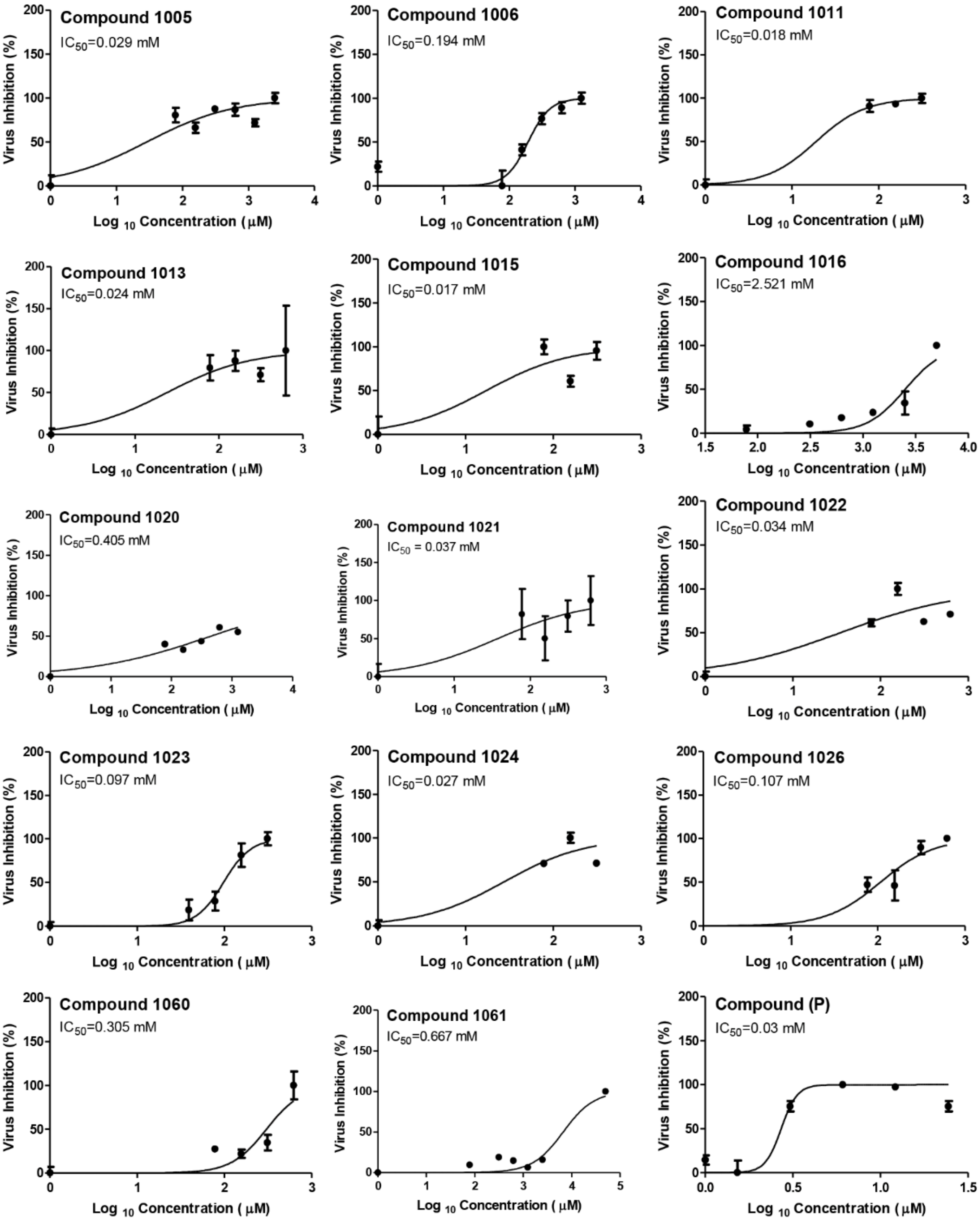

Figure S26: Antiviral activities against SARS-CoV-2

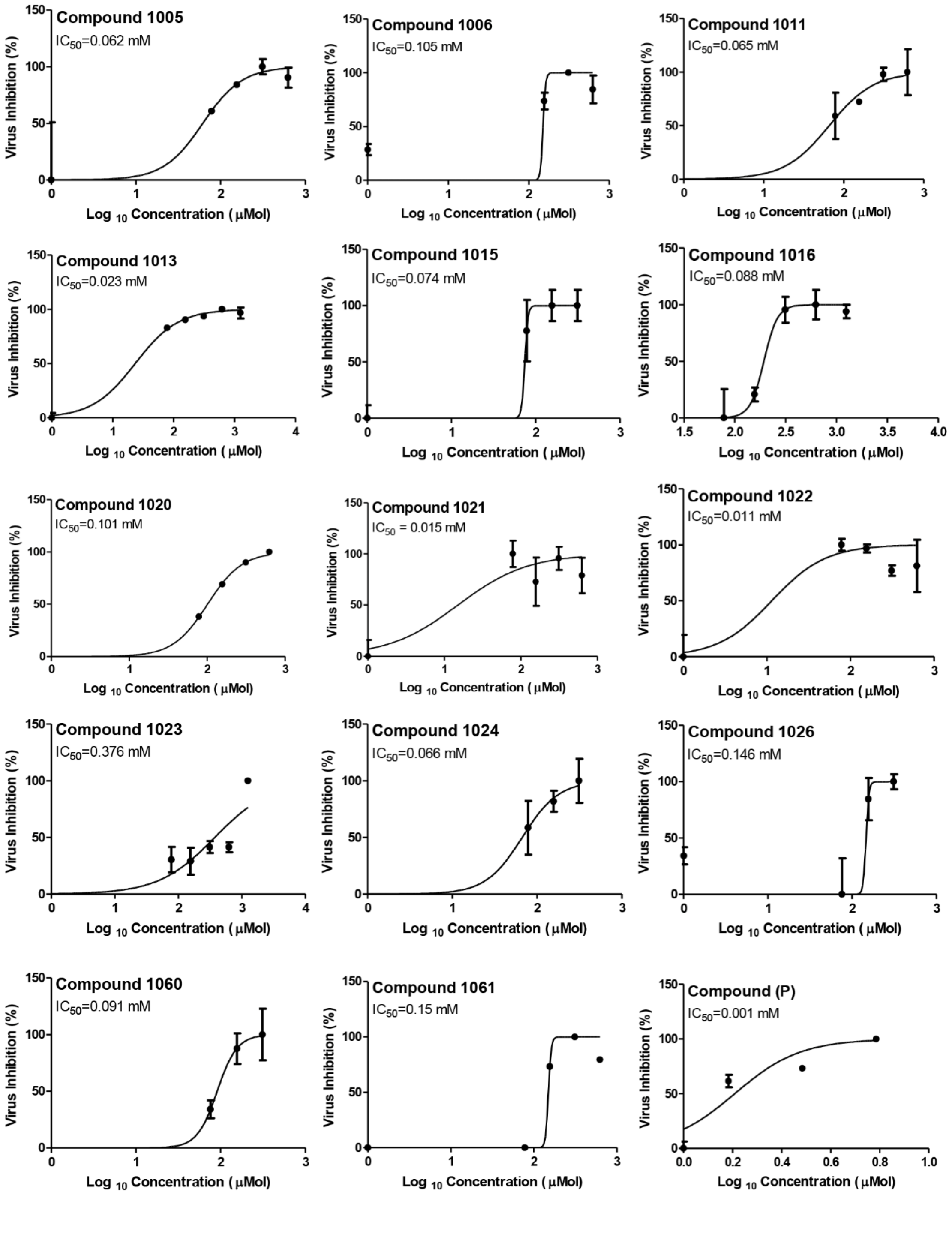

Figure S27: Antiviral activities against MERS-CoV

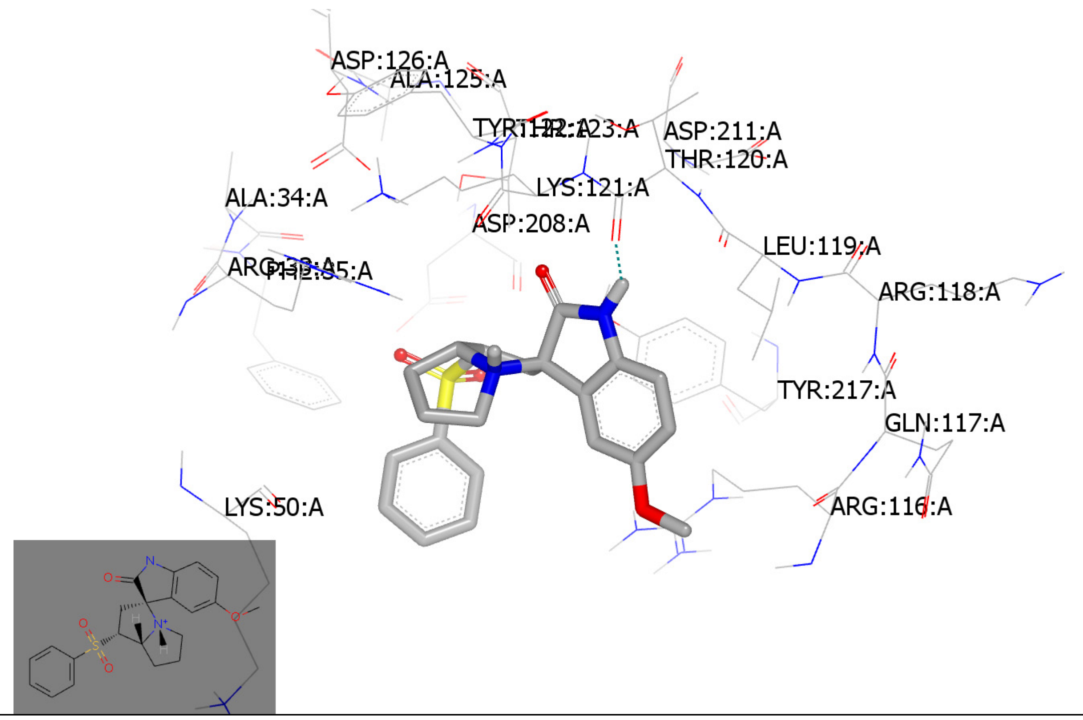

Figure S28: visual representation for compounds docked against (PDBID: 6m71) visualized by vida application : Compound **4h** binding mode and pose

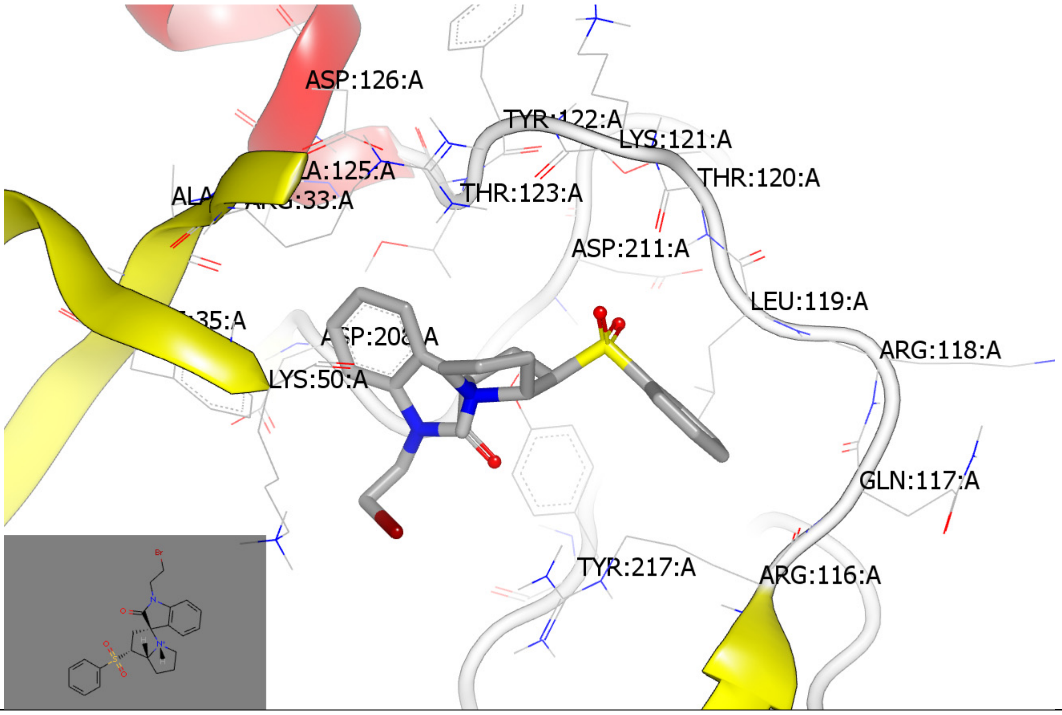

Figure S29: visual representation for compounds docked against (PDBID: 6m71) visualized by vida application**:** Compounds **4l** formed hydrophobic-hydrophobic interaction.
